# Predicting Treatment Failure With Sodium-Glucose Cotransporter-2 Inhibitors in People With Type 2 Diabetes: Novel Artificial Intelligence and Machine Learning Approach

**DOI:** 10.2196/85372

**Published:** 2026-05-20

**Authors:** Doyoung Kwak, Xi Tan, Yuanjie Liang, Caroline Swift, Chalak Muhammad, Xu Shi

**Affiliations:** 1 Texas A&M University, College Station, TX, United States; 2 Novo Nordisk Inc, 800 Scudders Mill Road, Plainsboro, NJ, 08536, United States; 3 University of Michigan, Ann Arbor, MI, United States

**Keywords:** sodium-glucose cotransporter-2 inhibitors, SGLT2i, type 2 diabetes, artificial intelligence, machine learning, treatment failure

## Abstract

**Background:**

The rate of treatment failure with sodium-glucose cotransporter-2 inhibitors (SGLT2i) is high among individuals with type 2 diabetes (T2D). Accurately predicting SGLT2i treatment failure is important for improving the clinical management of T2D.

**Objective:**

The study aimed to use machine learning (ML) models to identify factors predicting treatment failure with SGLT2i in T2D and to evaluate model performance.

**Methods:**

This retrospective observational cohort study included adults with T2D treated with SGLT2i (2016-2024). The primary outcome was overall treatment failure with SGLT2i during follow-up (≥180 days after SGLT2i initiation). The secondary outcome was subtypes of treatment failure with SGLT2i (treatment discontinuation, failure with action, and inertial failure) or nonfailure, which was defined as not meeting the definition for one of the failure subtypes. Variables potentially associated with treatment failure were assessed during the year before SGLT2i treatment initiation (analysis 1) and the year before SGLT2i treatment failure (analysis 2). Using these variables, ML models—logistic regression (LR), multilayer perceptron (MLP), extreme gradient boosting (XGBoost), and Transformer—were used to identify significant predictors of the outcomes. Model performance metrics (accuracy, area under the curve, precision, recall, and *F*_1_-score) were calculated. Using Shapley Additive Explanations methodology, key features were identified based on their impact on model predictions. LR and Transformer models using key features were further evaluated for their potential to support the development of a risk score for predicting treatment failure with SGLT2i.

**Results:**

Among all individuals in the study (N=62,222), 71% (n=44,156) had treatment failure with SGLT2i. Across subtypes, failure with action (n=23,839, 38.3%) was more common than treatment discontinuation (n=16,449, 26.4%) and inertial failure (n=3868, 6.2%). Model performance was moderate in both analyses. In analysis 1, the accuracy ranged from 0.72 to 0.73 for predicting overall treatment failure and from 0.56 to 0.57 for predicting the subtype of treatment failure. In analysis 2, the accuracy ranged from 0.74 to 0.75 for predicting overall treatment failure and from 0.61 to 0.63 for predicting the subtype of treatment failure. XGBoost, MLP, and Transformer models showed small improvements compared with LR. Using the top 9 key features identified from the Shapley Additive Explanations analysis, the Transformer model performed similarly in accuracy and area under the curve to its counterpart using the full feature set.

**Conclusions:**

Performance across the LR, MLP, XGBoost, and Transformer models was moderate. The advanced ML models performed slightly better than LR. Overall, the results suggest that further model advancements and increased data availability are needed to better predict treatment failure with SGLT2i. The LR coefficients from the key features model may inform the development of a risk score to predict SGLT2i treatment failure. Accurate prediction could inform individualized treatment planning for individuals with T2D.

## Introduction

Type 2 diabetes (T2D), a chronic and progressive metabolic condition caused by inefficient production or use of insulin and identified by elevated blood glucose levels, affects more than 35 million adults in the United States [1]. Sodium-glucose cotransporter-2 inhibitors (SGLT2i), a commonly prescribed therapy to reduce blood glucose levels in people with T2D, promote the excretion of glucose and reduce its reabsorption into the bloodstream [2]. SGLT2i also have additional benefits beyond glycemic control, including renal and cardiovascular benefits [3-5]. The American Diabetes Association recommends SGLT2i for individuals with T2D and chronic kidney disease (CKD) or kidney damage, as well as those with T2D and heart failure [5].

Despite the benefits of SGLT2i in managing T2D, treatment failure rates are high among individuals in real-world settings [6-8], particularly among those with comorbidities [9]. Treatment failure is a multifaceted problem, and there is currently no consensus on the definition of treatment failure with SGLT2i in people with T2D. Typical components of treatment failure may include discontinuation, switching to other glucose-lowering therapies (GLT), and treatment intensification [9,10]. Treatment failure with SGLT2i may also be characterized by not reaching the desired treatment goal after a certain amount of time or the attenuation of SGLT2i benefits over time [11].

Previous research has identified several factors associated with SGLT2i treatment discontinuation, including older age, specific comorbidities (eg, ischemic heart disease, chronic obstructive pulmonary disease, CKD, and cancer), the type of other GLT used at baseline, adverse effects, lack of efficacy, and financial reasons [7,9,12]. However, the literature lacks a comprehensive assessment of the factors contributing to treatment failure with SGLT2i or its different components, and identifying individuals at higher risk of treatment failure with SGLT2i can be clinically challenging. Identifying individuals for whom SGLT2i treatment is likely to fail has implications for treatment planning and clinical management and, ultimately, the achievement of treatment goals. The prediction of treatment failure with SGLT2i would enable health care professionals to take actions to reduce the likelihood of treatment failure or plan for possible failure based on the individual’s unique set of characteristics and circumstances.

Advanced machine learning (ML) models could potentially improve the prediction of treatment failure with SGLT2i in individuals with T2D. By analyzing data on many demographic characteristics, clinical parameters, laboratory values, and treatment histories, advanced ML models may offer advantages over conventional statistical methods for identifying factors and patterns that predict complex, multifaceted outcomes. In contrast to traditional techniques, which often rely on expert-driven a priori feature selection, modern algorithms such as gradient boosting and deep neural networks can automatically screen hundreds of candidate variables, rank their relative importance, and surface nonobvious predictors with minimal manual effort [13,14]. Additionally, by incorporating a broad array of variables, advanced ML models can potentially improve the accuracy and precision of outcome predictions as their flexible architectures capture complex nonlinear relationships and high-order interactions that conventional techniques, such as standard regression frameworks, struggle to represent without extensive handcrafting.

This study aimed to develop and internally validate ML models to predict treatment failure with SGLT2i among people with T2D using linked electronic health records (EHRs) and claims data. Specifically, we aimed to use ML models to predict treatment failure with SGLT2i and its subtypes.

## Methods

### Study Design and Data Source

This retrospective observational cohort study included adults with T2D treated with SGLT2i. Data were obtained from Optum deidentified Market Clarity Data (Optum Market Clarity), comprising EHRs linked to medical and pharmacy claims from Optum-affiliated payers and additional third-party claims. The database contains records on more than 86 million US people and is considered nationally representative of the US population.

### Ethical Considerations

This study was conducted in accordance with the Declaration of Helsinki of 1975 and its subsequent revisions. The study database is Health Insurance Portability and Accountability Act (HIPAA)–compliant and deidentified, and data were analyzed without reidentification of or contact with study participants. Because this study used only previously collected, deidentified data, informed consent from study participants was not necessary. The WCG Institutional Review Board determined this study to be exempt under 45 Code of Federal Regulations (CFR) § 46.104(d)(4) [15].

### Data Selection

**Figure 1.**
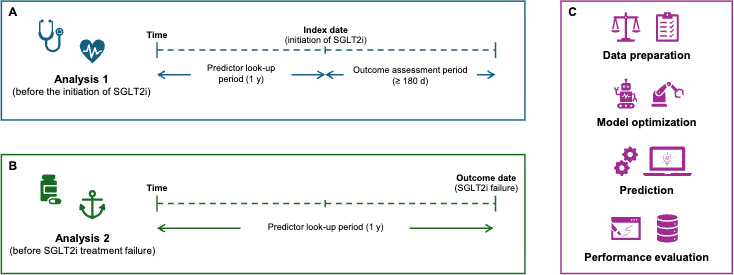
Study schematics. (A) Schematic of analysis 1; (B) schematic of analysis 2; and (C) schematic of data preparation, optimization, prediction, and evaluation steps. SLGT2i: sodium-glucose cotransporter-2 inhibitors; d, days; y, year;

The study period ran from January 1, 2016, through September 30, 2024. The index date was defined as the date of the first eligible SGLT2i pharmacy claim. The period for identifying eligible individuals for the study ran from December 31, 2016, through April 3, 2024, to include 1 year before the index date and a 180-day outcome assessment period (the interval from the index date to the end of the follow-up). Individuals were followed from the index date to the end of continuous claims enrollment, death, or the end of the study period, whichever occurred first. Two analyses, corresponding to two predictor lookup periods, were conducted to examine predictors of treatment failure with SGLT2i over two periods: (1) the year before SGLT2i treatment initiation (ie, analysis 1; Figure 1); and (2) the year before treatment failure with SGLT2i (ie, analysis 2; Figure 1). It was important to assess both time periods for predictors, as each can offer distinct, yet complementary, information. Analysis 1 focuses on predictors that can inform clinical decision-making before the initiation of treatment, whereas analysis 2 focuses on predictors more proximal to treatment failure, potentially informing clinical decision-making after treatment initiation.

### Inclusion and Exclusion Criteria

Individuals were eligible for inclusion in the study if they were newly initiated on an SGLT2i treatment (bexagliflozin, canagliflozin, dapagliflozin, empagliflozin, and ertugliflozin) between December 31, 2016, and April 3, 2024, and had 2 or more diagnoses of T2D on 2 or more distinct days during the study period, with the first T2D diagnosis on or before the index date. Eligible individuals also had continuous claims enrollment in the predictor lookup and outcome assessment periods, were aged 18 years or older on the index date, had 1 or more glycated hemoglobin (HbA_1c_) value ≥7% during the baseline period or on the index date, and had 1 or more HbA_1c_ value from 180 days after the index date until the end of follow-up. Individuals were excluded if they had 1 or more diagnoses of type 1 diabetes during the study period, had missing data for sex, initiated another new GLT drug class on the index date, used any glucagon-like peptide-1 receptor agonist (GLP-1 RA) obesity medications during the study period, had evidence of pregnancy, or had CKD stage 5 or end-stage kidney disease during the baseline period or on the index date (Figure S1 in Multimedia Appendix 1).

### Outcomes

The study outcome was treatment failure with SGLT2i, adopted from our previous work [8], which was defined as the occurrence of any of the following events during the outcome assessment period: initiation of a new GLT class (“failure with action”), discontinuation of SGLT2i (“discontinuation”), or HbA_1c_ not under control (“inertial failure”). Initiation of a new GLT class was defined as any pharmacy claim for a new GLT class (insulin, thiazolidinediones, sulfonylureas, GLP-1 RA, biguanides, meglitinides, alpha-glucosidase inhibitors, or dipeptidyl peptidase 4 inhibitors) during the follow-up period, including either switching to or adding a new GLT class. Discontinuation of SGLT2i was defined as a gap of 90 or more days for any SGLT2i during the follow-up period. Switching within an SGLT2i class was not considered discontinuation. HbA_1c_ was considered uncontrolled if 2 or more consecutive HbA_1c_ laboratory values were 8% or more on distinct days (≥90 days apart) between the index date plus 180 days and the end of the follow-up period. Nonfailure was defined as not meeting the definition for 1 of the failure subtypes. The primary outcome in this study was a binary variable for the occurrence of treatment failure with SGLT2i (treatment failure did or did not occur). The secondary outcome was a multiclass variable for the subtypes of treatment failure—failure with action, treatment discontinuation, or inertial failure. Additional details regarding the definitions of treatment failure with SGLT2i have been previously described [8].

### Predictors

Potential predictors of treatment failure with SGLT2i included index year, demographic characteristics (age, sex, race, and geographic region), clinical characteristics (BMI [<25, 25‐29.9, 30‐34.9, 35‐39.9, or ≥40 kg/m^2^], kidney function and CKD (stage 1, 2, 3, or 4), time from first observed T2D diagnosis to the index date), laboratory tests (HbA_1c_, low-density lipoprotein cholesterol, high-density lipoprotein cholesterol, very low-density lipoprotein cholesterol, total cholesterol, triglycerides, and C-reactive protein), and vital signs (systolic and diastolic blood pressure measurements; Table S1 in Multimedia Appendix 1). The following baseline comorbidities were also included: atrial fibrillation, ischemic heart disease, deep vein thrombosis or pulmonary embolism, hypertension, dyslipidemia, components of atherosclerotic cardiovascular disease, myocardial infarction, other coronary heart disease, peripheral artery disease, metabolic dysfunction–associated steatohepatitis or metabolic dysfunction–associated steatotic liver disease, obesity, anxiety, depression, asthma, musculoskeletal pain, and osteoarthritis. Other predictors included the Charlson Comorbidity Index adjusted without diabetes [16,17], the Diabetes Complications Severity Index [18,19], tobacco smoking status, GLT use, selected non-GLT treatment use, health care resource utilization (all-cause and T2D-related hospitalizations and emergency department visits), and health care costs (for all-cause and T2D-related hospitalizations and emergency department visits). Detailed information regarding predictor construction can be found in Tables S2-S4 in Multimedia Appendix 1.

### Analysis

#### Model Overview and Performance Evaluation

For analyses 1 and 2, descriptive statistics (mean and SD for continuous variables and counts and percentages for categorical variables) were calculated for demographic characteristics, baseline comorbidities, clinical characteristics, laboratory tests, vital signs, GLT use, selected non-GLT treatment use, health care resource utilization, and health care costs. For both analyses, we evaluated the performance of 4 models—logistic regression (LR), multilayer perceptron (MLP), extreme gradient boosting (XGBoost), and Transformer—to predict treatment failure with SGLT2i. LR, a traditional statistical method known for its simplicity and interpretability, is suitable for binary classification tasks [20]. MLP, XGBoost, and Transformer are advanced ML models. MLP is a feedforward artificial neural network useful for capturing nonlinear relationships through hidden layers [21]. XGBoost is a gradient boosting framework known for its efficiency and accuracy in handling structured data and managing feature interactions [22]. Transformer leverages self-attention mechanisms to process data efficiently and capture complex patterns in diverse datasets [23]. For all analyses, we split the data with 80% as a training set (including model optimization) and 20% as a test set for internal validation. After training and optimization on the training set, we used the test set to evaluate the performance of each model and generate confusion matrices [24]. Model performance was evaluated based on accuracy, receiver operating characteristic area under the curve (ROC AUC), precision, recall, and *F*_1_-score [25,26]. Precision, recall, and *F*_1_-score were computed for the positive class for the binary outcome, and support-weighted averaging was used for the multiclass outcome to account for class imbalance. Analyses were conducted in Python (version 3.10; Python Software Foundation) using *scikit-learn*, XGBoost, and PyTorch, along with supporting libraries such as *NumPy* and *Pandas*.

#### Data Preparation and Handling of Missing Data

An illustration of the data preparation and processing workflow for each model (LR, MLP, XGBoost, and Transformer) is provided in Figure S2 in Multimedia Appendix 1. In preparing the dataset for the LR model, categorical variables were factorized and transformed into integers based on their unique categories. Numerical variables were normalized using MinMax scaling and centered at the midpoint before model fitting, ensuring all variables were on a comparable scale. Missing values were imputed with 0 on the transformed feature scale, thereby providing a neutral or reference value; because the data were transformed before imputation, an imputed 0 did not indicate an actual clinical measurement of 0. Missing values across all variables (categorical and numerical) were filled with 0 as a consistent approach to minimize their impact on model parameters.

For the MLP model, categorical variables were embedded into vectors, and missing values were filled with 0. Numerical variables were normalized using MinMax scaling. Because a significant number of numerical variables naturally had 0 values, missing numerical values were not filled with 0. To distinguish between actual 0 values and missing numerical values, a masking strategy was used to flag missing data, with each numerical vector concatenated with a binary mask to indicate missingness. This approach ensured that data abnormalities were managed without distortion and facilitated the MLP model’s ability to integrate both categorical embeddings and numerical inputs efficiently.

For the XGBoost model, categorical variables in the dataset were converted to a string format to ensure consistent handling, using XGBoost’s built-in method of setting “unknown” values to missing. XGBoost inherently handles missing data by learning default directions for nodes in decision trees and assigning optimal paths for instances with missing values during training. This approach simplifies data preparation and maintains performance without requiring imputation of missing values.

For the Transformer model, categorical variables were processed through embeddings, converting them into dense vector representations, with any missing values filled with 0. As with the MLP model, numerical variables were normalized using MinMax scaling, and missing values were handled by creating a mask to indicate the presence of missing data. This approach to missingness also facilitated the Transformer’s ability to use information on both missing and nonmissing values effectively. By integrating positional encoding, the Transformer model encapsulates categorical and numerical features and their complex interactions, enhancing its predictive capabilities on tabular data.

In routinely collected EHRs or claims data, missing data are often informative because they are driven by care processes rather than random measurement error [27]. We did not use multiple imputation to impute missing data for any model because multiple imputation relies on strong assumptions (eg, missing-at-random) and a well-specified imputation model. Applying multiple imputation indiscriminately when missingness is structurally driven by routine care may introduce bias or clinically implausible values [28].

#### Parameterization

For the MLP and Transformer models, hyperparameter optimization, including tuning learning rate, batch size, number of epochs, and architecture, was conducted using a representative subset of 3000 individuals from the training dataset. This subset of 3000 individuals was randomly sampled from the training set to reduce the substantial computational burden associated with hyperparameter optimization for advanced ML models. The optimized hyperparameters were then applied to train the full dataset [29]. For the XGBoost model, optimization was performed through 5-fold cross-validation on the training set, evaluating accuracy and *F*_1_-scores across combinations of hyperparameters (eg, gamma, maximum depth, number of estimators, and learning rate). The optimal set of hyperparameters was selected based on cross-validation results, and the final model was retrained on the full training data before evaluation on the test set. The Appendix provides details on the optimization strategy (Table S2 in Multimedia Appendix 1), optimized hyperparameters (Table S3 in Multimedia Appendix 1), and final training iterations or epochs on the full training split (Table S4 in Multimedia Appendix 1) for each model. The same hyperparameter sets and optimization strategies were applied in analyses 1 and 2.

#### Feature Importance

Feature importance scores were derived to identify the contribution of individual variables in predicting SLGT2i treatment failure. Model-specific techniques were used to assess feature importance for each model. In the LR model, we compared the absolute values of coefficients and sorted them by magnitude to determine each feature’s relative influence. In the XGBoost model, the model’s internal function was used for importance calculation, focusing on metrics such as gain, cover, and frequency. “Gain” indicates the contribution of a feature to the model’s predictive capabilities, “cover” assesses the number of observations affected by splits on the feature, and “frequency” counts the occurrences of a feature in decision trees, thereby revealing the feature’s significance. In the Transformer model, a Shapley Additive Explanations (SHAP) analysis was conducted to interpret feature importance [30]. SHAP attributes the contribution of each feature according to its impact on model predictions. To enhance clinical utility, defined as the extent to which a predictive model informs clinical decision-making and actions to improve patient care, and support individualized treatment strategies, we identified key features based on their importance values in the best models. We ranked candidate predictors by their mean absolute SHAP value (global importance) and then selected features with clinical utility for further analysis to inform the development of a risk score or algorithm to predict treatment failure of SGLT2i. Upon conducting model comparisons, it was observed that the Transformer model performed slightly better than the other models while maintaining stability. Therefore, we focused on the Transformer model for key feature identification.

## Results

### Baseline Characteristics

A total of 62,222 individuals with T2D who initiated treatment with SGLT2i during the study period were included in the analyses (Tables S5 and S6 in Multimedia Appendix 1). More than half of the sample (n=35,487, 57%) was male, the mean (SD) age was 62.7 (12.0) years, and most individuals were White (n=44,774, 72%). Most individuals had commercial insurance (n=27,378, 44%) or Medicare (n=29,882, 48%). The most common comorbidities were hyperlipidemia (n=50,851, 81.7%), hypertension (n=50,681, 81.5%), and obesity (n=28,374, 45.6%; Table S5 in Multimedia Appendix 1). Overall, 71% (n=44,156) of individuals in the study experienced treatment failure with SGLT2i. When subtypes were assessed, failure with action (n=23,839, 38.3%) was more common than discontinuation (n=16,449, 26.4%) and inertial failure (n=3868, 6.2%; Table S5 in Multimedia Appendix 1).

### Features Analysis and Model Evaluation Results

**Figure 2.**
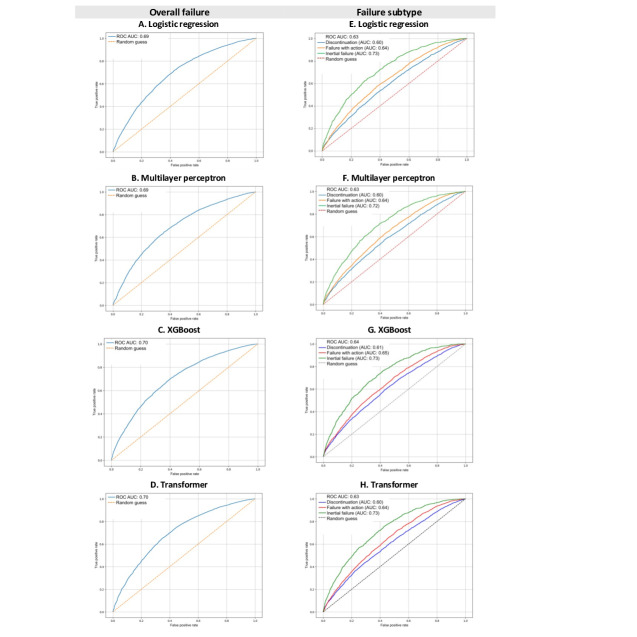
Receiver operating characteristic (ROC) curves (analysis 1). AUC: area under the curve; XGBoost: extreme gradient boosting. Models used to predict overall failure: (A) logistic regression; (B) multilayer perceptron; (C) XGBoost; (D) Transformer. Models used to predict failure subtype: (E) logistic regression; (F) multilayer perceptron; (G) XGBoost; (H) Transformer.

**Figure 3.**
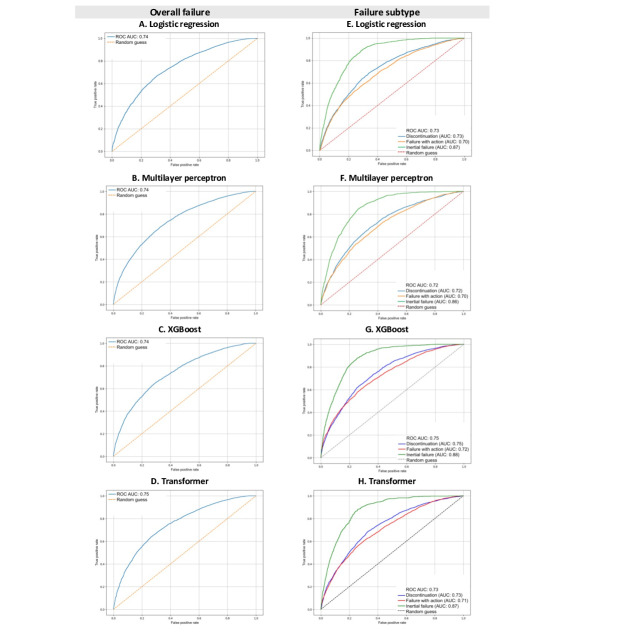
Receiver operating characteristic (ROC) curves (analysis 2). AUC: area under the curve; XGBoost: extreme gradient boosting. Models used to predict overall failure: (A) logistic regression; (B) multilayer perceptron; (C) XGBoost; (D) Transformer. Models used to predict failure subtype: (E) logistic regression; (F) multilayer perceptron; (G) XGBoost; (H) Transformer.

In both analyses 1 and 2, using the full dataset, model performance was moderate overall (Table 1). Compared with the LR model, the Transformer, XGBoost, and MLP models showed small improvements (Table 1). In analysis 1, accuracy across models ranged from 0.72 to 0.73 for overall failure or not (binary outcome) and from 0.56 to 0.57 for the subtypes of failure (multiclass outcome). ROC AUC across models ranged from 0.69 to 0.70 for overall failure and from 0.63 to 0.64 for the failure subtypes (Figure 2). In analysis 2, accuracy across models ranged from 0.74 to 0.75 for overall failure and from 0.61 to 0.63 for the failure subtypes. ROC AUC ranged from 0.74 to 0.75 for overall failure and from 0.72 to 0.75 for the failure subtypes (Figure 3). Similar patterns were observed for precision, recall, and *F*_1_-score estimates in both analyses 1 and 2. Notably, models developed for analysis 2 consistently outperformed those from analysis 1, particularly in the subtypes of the failure prediction task (Table 1).

**Table 1.** Performance metrics for models used to predict treatment failure with SGLT2i^a^ among people with type 2 diabetes.

Model	Accuracy	ROC AUC^b^	Precision	Recall	*F*_1_-score
Analysis 1^c^					
Overall failure (failure vs not)					
LR^d^	0.72	0.69	0.73	0.96	0.83
MLP^e^	0.72	0.69	0.74	0.92	0.82
XGBoost^f^	0.73	0.70	0.74	0.95	0.83
Transformer	0.73	0.70	0.73	0.96	0.83
Subtype of failure (discontinuation, failure with action, and inertial failure) or nonfailure					
LR	0.56	0.63	0.53	0.56	0.50
MLP	0.56	0.63	0.50	0.56	0.46
XGBoost	0.57	0.64	0.53	0.57	0.50
Transformer	0.57	0.63	0.56	0.57	0.49
Analysis 2^c^					
Overall failure (failure vs not)					
LR	0.74	0.74	0.76	0.93	0.84
MLP	0.74	0.74	0.76	0.93	0.84
XGBoost	0.74	0.74	0.77	0.92	0.84
Transformer	0.75	0.75	0.77	0.91	0.83
Subtype of failure (discontinuation, failure with action, and inertial failure) or nonfailure					
LR	0.61	0.73	0.60	0.61	0.59
MLP	0.61	0.72	0.60	0.61	0.59
XGBoost	0.63	0.75	0.62	0.63	0.61
Transformer	0.61	0.73	0.61	0.61	0.60

a SGLT2i: sodium-glucose cotransporter-2 inhibitor.

b ROC AUC: receiver operating characteristic area under the curve.

c Analyses were conducted to examine predictors of treatment failure with SGLT2i over two periods: (1) the year before SGLT2i treatment initiation (ie, analysis 1) and (2) the year before treatment failure with SGLT2i (ie, analysis 2).

d LR: logistic regression.

e MLP: multilayer perceptron.

f XGBoost: extreme gradient boosting.

### Feature Importance and Key Features Analysis

**Figure 4.**
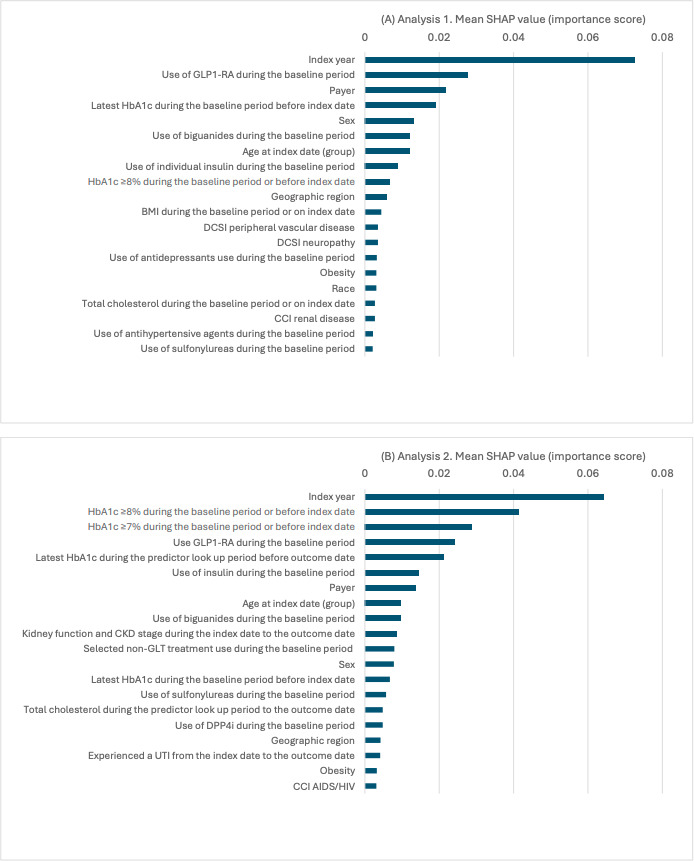
Transformer model in analysis 1 and analysis 2: feature importance bar plot from Shapley Additive Explanations (SHAP) analysis. (A) Analysis 1. Mean SHAP Value (Importance Score); (B) Analysis 2. Mean SHAP Value (Importance Score). CCI: Charlson Comorbidity Index; CKD: chronic kidney disease; DCSI: Diabetes Complication Severity Index; DPP4i: dipeptidyl peptidase 4 inhibitor; GLP-1 RA: glucagon-like peptide-1 receptor agonist; GLT: glucose-lowering therapy; HbA_1c_: glycated hemoglobin; UTI: urinary tract infection.

The SHAP analysis based on the Transformer model uncovered consistent feature importance patterns across analyses 1 and 2. Index year, HbA_1c_, and use of GLP-1 RA at baseline exhibited the highest importance scores in both analyses. Additionally, comparative examination of mean SHAP values and visualizations for the remaining individual features revealed similarities across the 2 analyses, with nuanced variations in the relative magnitude of these features. Analysis 2 also identified features after SGLT2i initiation, such as having experienced a urinary tract infection (Figure 4).

We identified key features for further analysis to support the development of a risk score or algorithm to predict treatment failure with SGLT2i. Because the index year, regarded as a proxy for follow-up time, may not be useful in clinical practice given the study period of this particular analysis and its potential limited utility in clinical decision-making, we excluded it from further analysis of key features. We tested both Transformer and LR models to evaluate their effectiveness using the remaining 9 top features (Figure 4): HbA_1c_≥8% during the baseline period or before the index date, use of GLP-1 RA during the baseline period; HbA_1c_≥7% during the baseline period or before the index date, use of insulin during the baseline period; and latest HbA_1c_ during the predictor lookup period before the outcome date, payer type, use of biguanides during the baseline period, age at index date (group), and kidney function and CKD stage during the index date to the outcome date. The number of key features was not determined by a statistical SHAP threshold; rather, it was chosen pragmatically to support clinical utility by limiting the number of required inputs and enabling a parsimonious model that could be operationalized as a simple risk score. Using the 9 selected key features, the Transformer model had slightly higher accuracy (0.75 vs 0.74) and recall (0.98 vs 0.97) and the same ROC AUC (0.70), precision (0.75), and *F*_1_-score (0.85) compared with the LR model (Table 2). The coefficients from the LR model with the 9 key features are presented in Table 3 and may inform further development of a risk score or algorithm to conveniently predict treatment failure with SGLT2i in routine clinical practice.

**Table 2.** Performance from the logistic regression and Transformer models with 9 key features.

Model	Accuracy	ROC AUC^a^	Precision	Recall	*F*_1_-score
LR model with 9 key features	0.74	0.70	0.75	0.97	0.85
Transformer model with 9 key features	0.75	0.70	0.75	0.98	0.85

a ROC AUC: receiver operating characteristic area under the curve.

**Table 3.** The coefficients from the logistic regression (LR) model with the 9 key features.

Key feature	LR coefficient (log odds)
HbA_1c_^a^ ≥8% during the baseline period or before index date	0.89
Use of GLP-1 RA^b^ during the baseline period	–0.50
HbA_1c_ ≥7% during the baseline period or before index date	–0.40
Use of insulin during the baseline period	–0.33
Latest HbA_1c_ during the predictor lookup period before outcome date	0.16
Payer	–0.15
Use of biguanides during the baseline period	0.14
Age at index date (group)	–0.02
Kidney function and CKD^c^ stage from the index date to the outcome date	0.02
Constant (intercept)	–0.49

a HbA_1c_: glycated hemoglobin.

b GLP-1 RA: glucagon-like peptide-1 receptor agonist.

c CKD: chronic kidney disease.

## Discussion

This real-world, observational study showed that treatment failure with SGLT2i is prevalent among people with T2D in the United States, which is consistent with our previous work [8]. We found that ML models produce modest performance improvements relative to traditional LR for predicting treatment failure with SGLT2i. Furthermore, we identified 9 predictors that may be particularly influential in predicting treatment failure with SGLT2i, including those readily available in EHR for individuals with T2D, such as HbA_1c_ levels, use of GLT, and kidney function. This study’s strengths included using a nationally representative US database with a large population and examining predictors in 2 reference periods—the year before treatment initiation with SGLT2i and the year before treatment failure with SGLT2i. This study also used advanced ML approaches to capture complex, nonlinear relationships and automatically identify important predictors from high-dimensional clinical data. Feature importance analysis was conducted to better explain the prediction models and enhance their utility in clinical practice.

This study provides important information for health care professionals on the high treatment failure rate with SGLT2i among people with T2D in real-world settings and the key predictors that may increase the risk of treatment failure. Overall, across analyses 1 and 2, model performance was moderate [31,32]. Although improvement in model performance is desirable, the findings suggest that advanced ML models, including MLP, XGBoost, and Transformer, can learn relevant patterns from the data. Furthermore, in analyses 1 and 2, these advanced ML models showed modest improvements in performance metrics over traditional LR, indicating a slightly better ability to capture nuanced patterns. The superior model performance in analysis 2 was likely due to postindex data providing information more proximal to treatment failure with SGLT2i, enabling more precise predictions. Having information post-SGLT2i initiation (index date), especially on potential SGLT2i treatment-related adverse events, may help boost predictive accuracy.

Building on the analyses with the full feature set, we examined the LR and Transformer models using a reduced set of 9 features selected based on importance scores. Both models demonstrated comparable performance, with scores reaching moderate levels overall. Notably, these models performed similarly to those using the full feature set, underscoring the potential utility of parsimonious models incorporating influential predictors. In the context of T2D and SGLT2i treatment, feature ranking facilitates the identification of the most influential predictors for determining treatment failure with SGLT2i. From a clinical perspective, ML and strategic feature selection could empower health care professionals by helping them identify individuals at the highest risk of treatment failure and other treatment-related outcomes, such as medication nonadherence, for further treatment planning or intervention [33-35].

Although ML models hold promise for clinical prediction, data quality issues, the need for large datasets, costs, and implementation challenges in real-world settings impede the uptake of ML-based prediction models in clinical practice [36]. LR is commonly used to predict clinical outcomes, including treatment failure, in clinical practice settings due to its familiarity among health care professionals and its ability to handle binary outcomes. Coefficients from LR models offer insight into the relationship between a set of predictors and the likelihood of treatment failure. Coefficients from the LR model used in our study could inform the development of a future prediction tool to estimate the likelihood of treatment failure with SGLT2i among individuals with T2D. Until such a prediction tool is available, the key features identified in this study (eg, HbA_1c_≥7%, payer type, or use of insulin, biguanides, or GLP-1 RA before SGLT2i initiation) could be of value for clinical decision-making to reduce treatment failure among people with T2D.

Future research is needed to refine and validate ML models across populations and settings to increase the applicability of ML-based predictive results to clinical practice in T2D while addressing deficiencies in data quality and quantity [37]. Specifically, there is a need to increase the breadth of predictors to encompass health behaviors, family history, genetic information, social determinants of health, patient-reported outcomes, and comprehensive laboratory data. Concurrently, data quality must also be addressed to improve its accuracy and completeness, thereby enhancing the efficiency and utility of predictive analytics. Furthermore, although ML methodologies, including deep learning, have demonstrated efficacy in medical imaging, diagnosis, and early detection of disease or complications, their performance in predicting clinical outcomes is suboptimal. There is a need to develop ML models that can utilize complex, high-dimensional data to produce highly accurate, clinically relevant information [38-40]. Given the modest performance gains observed, LR remains a strong baseline for clinical deployment due to its superior interpretability. Our results also indicate that the current data and composite end point likely cap performance across models, while advanced architectures may provide meaningful value with richer, longitudinal, or multimodal inputs for tasks closer to the failure event, as suggested by the consistently better performance in analysis 2. Finally, the 9-feature model serves as a pragmatic bridge to clinical use by retaining comparable performance with far greater simplicity.

This study had some limitations. Although ML models can identify features strongly correlated with treatment failure with SGLT2i, these correlations do not necessarily imply causation. This is an inherent limitation of observational study design. Second, the datasets used to build and validate the models comprised administrative claims-linked EHR data, which were not collected for research purposes. These data may include potential inaccuracies in diagnostic coding, measurement error, and incomplete information on variables used in the predictive models. Different strategies for handling missing data may enable some models to use missingness patterns more directly than others, potentially influencing model performance. However, these strategies reflect inherent differences in model architecture and standard practice and are unlikely to have driven model performance because differences between advanced models and LR were small overall. Although explicit masking could confer an advantage for deep learning models, we observed only modest improvements. Given the large sample size and the substantial computational burden associated with training advanced models—particularly XGBoost and the Transformer—we used a fixed 80%/20% training/testing split for final model evaluation. Although performance metrics based on a single internal split may not fully capture variability across data partitions, this approach enabled consistent comparisons of performance. Regarding the design of predictors, we aimed at clinical interpretability, with conditions and drug utilization over a period of time summarized. A more complex design incorporating the longitudinal nature of predictors (ie, the timing and sequence) or more free-form predictors (eg, unlabeled diagnosis codes) may improve the predictive accuracy of some of the models but at a loss of clinical interpretation. Furthermore, the datasets used in this study did not contain data on certain factors that could be important for predicting treatment failure with SGLT2i, such as more granular clinical characteristics, behavioral factors, and social determinants of health. Furthermore, although this study’s focus was a parsimonious, clinically implementable single-risk score, subtype-specific models could further disentangle underlying mechanisms of treatment failure with SGLT2i, which could be an important avenue for future research. Finally, this study focused on the glycemic control indication for T2D and did not include other indications for SGLT2i (eg, cardiovascular disease).

This study found a high prevalence of treatment failure with SGLT2i among people with T2D. In predicting overall treatment failure and subtype, model performance was moderate. Models based on the examination of predictors in the year before treatment failure with SGLT2i performed better than models based on the examination of predictors in the year before SGLT2i initiation. The results suggest that further advancements in ML models and additional data are needed to enhance ML-based prediction of treatment failure with SGLT2i. Feature importance analysis may support the development of a risk score or algorithm to inform more timely individualized treatment planning for individuals with T2D.

## Supplementary material

10.2196/85372 Multimedia Appendix 1 Supplemental material.

## Abbreviations

CKD: chronic kidney disease
EHR: electronic health record
GLP-1 RA: glucagon-like peptide-1 receptor agonist
GLT: glucose-lowering therapy
HbA_1c_: glycated hemoglobin
HIPAA: Health Insurance Portability and Accountability Act
LR: logistic regression
ML: machine learning
MLP: multilayer perceptron
ROC AUC: receiver operating characteristic area under the curve
SGLT2i: sodium-glucose cotransporter-2 inhibitor
SHAP: Shapley Additive Explanations
T2D: type 2 diabetes
XGBoost: extreme gradient boosting
