# Supplementary material for: Predicting Treatment Failure With Sodium-Glucose Cotransporter-2 Inhibitors in People With Type 2 Diabetes: Novel Artificial Intelligence and Machine Learning Approach
Source: JMIR Diabetes. 2026 May 20;11:e85372. doi: 10.2196/85372 (PMC13189570; doi:10.2196/85372)
Supplement: Multimedia Appendix 1 [file diabetes-v11-e85372-s001.docx]

# Multimedia Appendix. Supplemental Material

**Title:** Predicting Treatment Failure With Sodium-Glucose Cotransporter-2 Inhibitors (SGLT2i) in People With Type 2 Diabetes: A Novel Artificial Intelligence and Machine Learning Approach

**Authors:**

Doyoung Kwak^1^, MS; Xi Tan^2^, PhD, PharmD; Yuanjie Liang^2^, MS; Caroline Swift^2^, PhD; Chalak Muhammad^2^, MD, MPH; Xu Shi^2^, PhD

**Affiliations:**

^1^Texas A&M University, College Station, TX, USA

^2^Novo Nordisk Inc., Plainsboro, NJ, USA

^3^University of Michigan, Ann Arbor, MI, USA

**Corresponding Author:**

Xi Tan, PhD, PharmD

Novo Nordisk Inc.

800 Scudders Mill Road

Plainsboro, NJ, 08536

United States

Phone: 1 609 366 4636

Email: [MXTZ@novonordisk.com](https://doi.org/10.1016/j.eswa.2021.115895)

**Schedule of Supplemental Material**

Plain language summary.

Appendix Table 1. Operational definitions of predictors.

Appendix Table 2. Optimization strategy by model.

Appendix Table 3. Hyperparameters optimized by model.

Appendix Table 4. Final training iterations/epochs on the full training split.

Appendix Table 5. Analysis 1: Baseline characteristics, overall and by treatment failure subtype.

Appendix Table 6. Analysis 2: Baseline characteristics, overall and by treatment failure subtype.

Appendix Figure 1. Study sample selection flow diagram.

Appendix Figure 2. Data preparation and processing diagram.

**Plain Language Summary**

People with type 2 diabetes (T2D) take medications to manage their condition. Health care professionals often prescribe sodium-glucose cotransporter-2 inhibitors, or SGLT2i, to people with T2D. These medications help control blood sugar levels and protect the heart and kidneys. However, these medications may not effectively control blood sugar levels for everyone with T2D. When treatment is not successful, this is called treatment failure. Treatment failure with SGLT2i is common among people with T2D. We wanted to learn more about the reasons for treatment failure and what methods could best predict these reasons. In this study, we looked at a large group of people with T2D. Statistical models, including those using machine learning, a type of artificial intelligence, are useful tools for analyzing large amounts of data. We used machine learning models to assess treatment failure with SGLT2i, including different kinds of treatment failure with SGLT2i (stopping treatment, starting a new treatment, or the treatment not working as expected). We also identified factors that predict treatment failure with SGLT2i. The study found that 7 in 10 people with T2D had treatment failure with SGLT2i. The machine learning models were moderately accurate in predicting treatment failure. Factors predicting treatment failure included the individual’s age, blood glucose level, type of health insurance, use of other medications to treat T2D, and how well their kidneys work. Although more research is needed, what we learned from this study could help improve the care of people with T2D. A risk score could eventually be created based on the predictors of treatment failure, which health care professionals could use to make individualized treatment plans.

**Appendix Table 1.** Operational definitions of predictors.

| **Predictors** | **Sources** | **Assessment period** | **Comments** |
| --- | --- | --- | --- |
| **Type 2 diabetes (T2D)** | Claims; ICD-10-CM codes | The whole study period | Inclusion criteria: had ≥2 claims of E11.X on ≥2 distinct days during the study period, with the first claims on or before the index date |
| **Demographics** | Claims | Analysis 1: one year before SGLT2i treatment initiation  Analysis 2: one year before treatment failure with SGLT2i |  |
| **Common comorbidities** | Claims; ICD-10-CM codes | Same as above | Tan X, Cao Y, Wang Y, Boland J, Guevarra M, Xie L, Song Y, Kwak MJ. Prevalence, treatment patterns, burden, and factors related to treatment failure with sodium-glucose cotransporter 2 inhibitor in adults with type 2 diabetes in the United States. Diabetes Metab Syndr. 2025 Jul;19(7):103281. |
| **ASCVD components including ischemic stroke, transient ischemic attack, other atherosclerotic cerebrovascular disease, myocardial infarction, peripheral artery disease, other coronary heart disease** | Claims; ICD-10-CM codes | Same as above | Tan X, Liang Y, Rajpura JR, Yedigarova L, Noone J, Xie L, Inzucchi S, de Havenon A. Once-weekly glucagon-like peptide-1 receptor agonists vs dipeptidyl peptidase-4 inhibitors: cardiovascular effects in people with diabetes and cardiovascular disease. Cardiovasc Diabetol. 2023 Nov 20;22(1):319. |
| **Charlson Comorbidity Index adjusted without diabetes** | Claims; ICD-10-CM codes | Same as above | Charlson ME, Pompei P, Ales KL, et al. A new method of classifying prognostic comorbidity in longitudinal studies: development and validation. J Chronic Dis. 1987;40(5):373–383. doi: 10.1016/0021-9681(87)90171-8**.**  Glasheen WP, Cordier T, Gumpina R, Haugh G, Davis J, Renda A. Charlson Comorbidity Index: ICD-9 Update and ICD-10 Translation. Am Health Drug Benefits. 2019 Jun-Jul;12(4):188-197. |
| **Diabetes Complications Severity Index** | Claims; ICD-10-CM codes | Same as above | Young BA, Lin E, Von Korff M, et al. Diabetes complications severity index and risk of mortality, hospitalization, and healthcare utilization. Am J Manag Care. 2008;14(1):15–23.  Glasheen WP, Renda A, Dong Y. Diabetes Complications Severity Index (DCSI)-Update and ICD-10 translation. J Diabetes Complications. 2017 Jun;31(6):1007-1013. |
| **Tobacco smoking status** | Claims; ICD-10-CM codes | Same as above | ICD-10 codes: F17.x, Z71.6, Z72.0 |
| **Kidney function and CKD stage** | Claims and EHR; ICD-10-CM codes, LOINC codes | Same as above | ICD-10 codes: (Stage 1: N18.1, Stage 2: N18.2, Stage 3: N18.3, Stage 4: N18.4, Stage 5: N18.5, N18.6).  eGFR-based staging required two eGFR values 90-365 days apart meeting the following cutoffs: Stage 1 or 2: ≥60; Stage 3: 30-59; Stage 4: 15-29; Stage 5: <15. The two eGFR values were treated as follows: when they corresponded to the CKD stage, the higher stage encompassed the lower stage. For instance, a stage 3 eGFR value is suitable for identifying both stage 2 and stage 3. |
| **HbA1c** | Claims and EHR; LOINC codes | Same as above; additionally, select the valid records closest to the SGLT2i treatment initiation date for Analysis 1 and closest to the treatment failure date for Analysis 2. | LOINC: 41995-2, 71875-9, 17855-8, 17856-6, 4548-4, 4549-2, 55454-3, 4637-5, 43150-2 |
| **BMI** | Claims and vital signs; LOINC codes and ICD-10-CM codes | Same as above; additionally, select the valid records closest to the SGLT2i treatment initiation date for Analysis 1 and closest to the treatment failure date for Analysis 2. | LOINC codes: 39156-5, 89270-3, 41909-3;  ICD-10 codes: Z68.2x, Z68.3x, Z68.4x |
| **LDL** | Claims and EHR; LOINC codes | Same as above; additionally, select the valid records closest to the SGLT2i treatment initiation date for Analysis 1 and closest to the treatment failure date for Analysis 2. | LOINC codes: 18262-6, 2089-1, 2090-9, 49132-4, 55440-2, 96259-7, 13457-7, 91105-7, 91106-5, 91107-3, 91108-1, 91109-9 |
| **HDL** | Claims and EHR; LOINC codes | Same as above; additionally, select the valid records closest to the SGLT2i treatment initiation date for Analysis 1 and closest to the treatment failure date for Analysis 2. | LOINC codes: 2085-9, 49130-8 |
| **VLDL** | Claims and EHR; LOINC codes | Same as above; additionally, select the valid records closest to the SGLT2i treatment initiation date for Analysis 1 and closest to the treatment failure date for Analysis 2. | LOINC codes: 13458-5, 2091-7 |
| **Total cholesterol** | Claims and EHR; LOINC codes | Same as above; additionally, select the valid records closest to the SGLT2i treatment initiation date for Analysis 1 and closest to the treatment failure date for Analysis 2. | LOINC codes: 2093-3 |
| **Triglycerides** | Claims and EHR; LOINC codes | Same as above; additionally, select the valid records closest to the SGLT2i treatment initiation date for Analysis 1 and closest to the treatment failure date for Analysis 2. | LOINC codes: 2571-8, 14927-8, 70218-3 |
| **C-reactive protein** | Claims and EHR ; LOINC codes | Same as above; additionally, select the valid records closest to the SGLT2i treatment initiation date for Analysis 1 and closest to the treatment failure date for Analysis 2. | LOINC codes: 1988-5 |
| **Systolic blood pressure** | Claims and vital signs; LOINC codes | Same as above; additionally, select the valid records closest to the SGLT2i treatment initiation date for Analysis 1 and closest to the treatment failure date for Analysis 2. | LOINC codes: 8480-6, 8459-0 |
| **Diastolic blood pressure** | Claims and vital signs; LOINC codes | Same as above; additionally, select the valid records closest to the SGLT2i treatment initiation date for Analysis 1 and closest to the treatment failure date for Analysis 2. | LOINC codes: 8462-4, 8453-3 |
| **Medications** | Pharmacy claims; drug generic names | Same as above |  |
| **Healthcare resource utilization** | Claims; ICD-10-CM codes | Same as above | Mean (SD):  ▪ Number of all-cause hospitalization  ▪ Number of all-cause ED visits  ▪ Number of T2D-related hospitalizations  ▪ Number of T2D-related ED visits |
| **Healthcare cost** | Claims; ICD-10-CM codes | Same as above | Mean (SD):  ▪ Cost of all-cause hospitalization  ▪ Cost of all-cause ED visits  ▪ Cost of T2D-related hospitalizations  ▪ Cost of T2D-related ED visits |

Abbreviations: ASCVD, atherosclerotic cardiovascular disease; BMI, body mass index; CKD, chronic kidney disease; ED, emergency department; EHR, electronic health records; HbA1c, glycated hemoglobin; HDL, high-density lipoprotein; ICD-10-CM, International Classification of Diseases, Tenth Revision, Clinical Modification; LDL, low-density lipoprotein; LOINC, Logical Observation Identifiers Names and Codes; SGLT2i, sodium-glucose cotransporter-2 inhibitors; T2D, type 2 diabetes; VLDL, very low-density lipoprotein.

**Appendix Table 2.** Optimization strategy by model.

| Model | Optimizer/Solver | Objective Function (Loss) | Scheduler/Other Settings |
| --- | --- | --- | --- |
| Transformer | Adam (lr=0.0001, weight_decay=1e-10) | CrossEntropyLoss (Binary: Weighted, Multi: Standard) | CosineAnnealingLR |
| MLP | Adam (lr=0.005) | CrossEntropyLoss | ReduceLROnPlateau |
| XGBoost | Tree Construction: hist | Binary: binary:logistic Multi: multi:softprob | Regularization: L2 (lambda), Gamma, Learning Rate: eta |
| LR | lbfgs | Log Loss | L2 Penalty (Standard) |

Abbreviations: LR, logistic regression; MLP, Multilayer Perceptrons; XGBoost, Extreme Gradient Boosting.

**Appendix Table 3.** Hyperparameters optimized by model.

| **Model** | **Hyperparameters** |
| --- | --- |
| LR | Not applicable |
| MLP | • Hidden layer size/width  • Dropout rate  • Batch size  • Learning rate |
| XGBoost | • Eta (learning rate/step size shrinkage)  • Gamma (minimum loss reduction required to make a further split)  • Maximum depth (maximum depth of each tree)  • Minimum child weight (minimum sum of instance weight needed in a child node)  • Maximum delta step (maximum step size allowed for weight updates)  • Lambda (L2 regularization term on weights)  • Maximum leaves (maximum number of leaves in a tree)  • Maximum bins (number of bins for histogram-based splitting) |
| Transformer | • Number of attention heads  • Number of Transformer layers  • Dropout rate  • Batch size  • Learning rate |

Abbreviations: LR, logistic regression; MLP, Multilayer Perceptrons; XGBoost, Extreme Gradient Boosting.

**Appendix Table 4.** Final training iterations/epochs on the full training split.

| Model | Task | Iterations used in final training |
| --- | --- | --- |
| LR | Binary | max_iter = 100 (default) |
| LR | Multiclass | max_iter = 100 (default) |
| MLP | Binary | epochs = 40 |
| MLP | Multiclass | epochs = 9 |
| XGBoost | Binary | boosting rounds = not set explicitly (library default: 100) |
| XGBoost | Multiclass | boosting rounds = not set explicitly (library default: 100) |
| Transformer | Binary | epochs = 1000 |
| Transformer | Multiclass | epochs = 1000 |

Abbreviations: LR, logistic regression; MLP, Multilayer Perceptrons; XGBoost, Extreme Gradient Boosting.

**Appendix Table 5.** Analysis 1: Baseline characteristics, overall and by treatment failure subtype.^a,b^

|  |  |  | **SGLT2i failure subtype** | | |  |
| --- | --- | --- | --- | --- | --- | --- |
| **Characteristics of individuals with T2D** | **Overall**  **sample N = 62,222** | **SGLT2i failure overall n = 44,156** | **Discontinuation group n = 16,449** | **Failure with action group n = 23,839** | **Inertial failure group n = 3,868** | **Nonfailure group n = 18,066** |
| **Demographics** |  |  |  |  |  |  |
| Age at index date (years), mean (SD) | 62.65 (11.96) | 62.03 (12.01) | 62.19 (12.19) | 62.07 (11.93) | 61.12 (11.65) | 64.17 (11.72) |
| Age at index date, n (%) |  |  |  |  |  |  |
| 18-34 | 740 (1.2) | 568 (1.3) | 231 (1.4) | 291 (1.2) | 46 (1.2) | 172 (1.0) |
| 35-44 | 3,787 (6.1) | 2,937 (6.7) | 1,146 (7.0) | 1,544 (6.5) | 247 (6.4) | 850 (4.7) |
| 45-54 | 11,068 (17.8) | 8,359 (18.9) | 2,997 (18.2) | 4,522 (19.0) | 840 (21.7) | 2,709 (15.0) |
| 55-64 | 18,355 (29.5) | 13,058 (29.6) | 4,690 (28.5) | 7,132 (29.9) | 1,236 (32.0) | 5,297 (29.3) |
| 65-74 | 17,583 (28.3) | 12,193 (27.6) | 4,643 (28.2) | 6,586 (27.6) | 964 (24.9) | 5,390 (29.8) |
| ≥75 | 10,689 (17.2) | 7,041 (15.9) | 2,742 (16.7) | 3,764 (15.8) | 535 (13.8) | 3,648 (20.2) |
| Sex, n (%) |  |  |  |  |  |  |
| Male | 35,487 (57.0) | 24,380 (55.2) | 9,228 (56.1) | 12,856 (53.9) | 2,296 (59.4) | 11,107 (61.5) |
| Race, n (%) |  |  |  |  |  |  |
| Black | 7,725 (12.4) | 5,671 (12.8) | 2,289 (13.9) | 2,837 (11.9) | 545 (14.1) | 2,054 (11.4) |
| Asian | 2,668 (4.3) | 1,798 (4.1) | 741 (4.5) | 886 (3.7) | 171 (4.4) | 870 (4.8) |
| White | 44,774 (72.0) | 31,602 (71.6) | 11,356 (69.0) | 17,466 (73.3) | 2,780 (71.9) | 13,172 (72.9) |
| Other/unknown | 7,055 (11.3) | 5,085 (11.5) | 2,063 (12.5) | 2,650 (11.1) | 372 (9.6) | 1,970 (10.9) |
| Ethnicity, n (%) |  |  |  |  |  |  |
| Hispanic | 7,139 (11.5) | 5,205 (11.8) | 2,086 (12.7) | 2,719 (11.4) | 400 (10.3) | 1,934 (10.7) |
| Not Hispanic | 49,914 (80.2) | 35,258 (79.8) | 12,876 (78.3) | 19,173 (80.4) | 3,209 (83.0) | 14,656 (81.1) |
| Unknown | 5,169 (8.3) | 3,693 (8.4) | 1,487 (9.0) | 1947 (8.2) | 259 (6.7) | 1476 (8.2) |
| Index year, n (%) |  |  |  |  |  |  |
| 2015/2016 | 6,765 (10.9) | 5,592 (12.7) | 2,004 (12.2) | 2,961 (12.4) | <5 (<1.0) | 1,173 (6.5) |
| 2017 | 7,026 (11.3) | 5,700 (12.9) | 2,198 (13.4) | 2,840 (11.9) | 627 (16.2) | 1,326 (7.3) |
| 2018 | 8,067 (13.0) | 6,347 (14.4) | 2,439 (14.8) | 3,207 (13.5) | 662 (17.1) | 1,720 (9.5) |
| 2019 | 8,652 (13.9) | 6,693 (15.2) | 2,468 (15.0) | 3,556 (14.9) | 701 (18.1) | 1,959 (10.8) |
| 2020 | 10,915 (17.5) | 7,968 (18.0) | 2,916 (17.7) | 4,376 (18.4) | 669 (17.3) | 2,947 (16.3) |
| 2021 | 11,224 (18.0) | 7,214 (16.3) | 2,731 (16.6) | 4,016 (16.8) | 676 (17.5) | 4,010 (22.2) |
| 2022 | 8,802 (14.1) | 4,402 (10.0) | 1,637 (10.0) | 2,699 (11.3) | 467 (12.1) | 4,400 (24.4) |
| 2023 | 771 (1.2) | 240 (0.5) | 56 (0.3) | 184 (0.8) | 66 (1.7) | 531 (2.9) |
| Geographic region, n (%) |  |  |  |  |  |  |
| Midwest | 23,140 (37.2) | 16,453 (37.3) | 5,929 (36.0) | 8,985 (37.7) | 1,539 (39.8) | 6,687 (37.0) |
| Northeast | 11,949 (19.2) | 8,228 (18.6) | 3,126 (19.0) | 4,327 (18.2) | 775 (20.0) | 3,721 (20.6) |
| South | 17,616 (28.3) | 12,717 (28.8) | 4,832 (29.4) | 6,896 (28.9) | 989 (25.6) | 4,899 (27.1) |
| West | 7,687 (12.4) | 5,420 (12.3) | 2,073 (12.6) | 2,893 (12.1) | 454 (11.7) | 2,267 (12.5) |
| Other/unknown | 1,830 (2.9) | 1,338 (3.0) | 489 (3.0) | 738 (3.1) | 111 (2.9) | 492 (2.7) |
| Payer, n (%) |  |  |  |  |  |  |
| Commercial | 27,378 (44.0) | 19,800 (44.8) | 7,017 (42.7) | 10,847 (45.5) | 1,936 (50.1) | 7,578 (41.9) |
| Medicaid | 4,127 (6.6) | 3,042 (6.9) | 1,253 (7.6) | 1,529 (6.4) | 260 (6.7) | 1,085 (6.0) |
| Medicare Advantage or Medicare | 29,882 (48.0) | 20,753 (47.0) | 7,954 (48.4) | 11,167 (46.8) | 1,632 (42.2) | 9,129 (50.5) |
| Other/unknown | 835 (1.3) | 561 (1.3) | 225 (1.4) | 296 (1.2) | 40 (1.0) | 274 (1.5) |
| **Baseline comorbidities**, n (%) |  |  |  |  |  |  |
| Atrial fibrillation | 5,492 (8.8) | 3,588 (8.1) | 1,364 (8.3) | 1,934 (8.1) | 290 (7.5) | 1,904 (10.5) |
| Ischemic heart disease | 16,354 (26.3) | 11,128 (25.2) | 4,274 (26.0) | 5,903 (24.8) | 951 (24.6) | 5,226 (28.9) |
| DVT and PE | 1,309 (2.1) | 911 (2.1) | 326 (2.0) | 504 (2.1) | 81 (2.1) | 398 (2.2) |
| Hypertension | 50,681 (81.5) | 35,739 (80.9) | 13,376 (81.3) | 19,186 (80.5) | 3,177 (82.1) | 14,942 (82.7) |
| Hyperlipidemia/dyslipidemia | 50,851 (81.7) | 35,855 (81.2) | 13,363 (81.2) | 19,289 (80.9) | 3,203 (82.8) | 14,996 (83.0) |
| ASCVD components including |  |  |  |  |  |  |
| Ischemic stroke | 1,811 (2.9) | 1,210 (2.7) | 476 (2.9) | 619 (2.6) | 115 (3.0) | 601 (3.3) |
| Transient ischemic attack | 1,059 (1.7) | 742 (1.7) | 304 (1.8) | 385 (1.6) | 53 (1.4) | 317 (1.8) |
| Other atherosclerotic  cerebrovascular disease | 4,148 (6.7) | 2,816 (6.4) | 1,150 (7.0) | 1,417 (5.9) | 249 (6.4) | 1,332 (7.4) |
| Myocardial infarction | 2,094 (3.4) | 1,431 (3.2) | 561 (3.4) | 744 (3.1) | 126 (3.3) | 663 (3.7) |
| Other coronary heart disease | 15,060 (24.2) | 10,248 (23.2) | 3,951 (24.0) | 5,418 (22.7) | 879 (22.7) | 4,812 (26.6) |
| Peripheral artery disease | 6,989 (11.2) | 4,771 (10.8) | 1,887 (11.5) | 2,487 (10.4) | 397 (10.3) | 2,218 (12.3) |
| MASH/MAFLD | 5,070 (8.1) | 3,651 (8.3) | 1,364 (8.3) | 2,056 (8.6) | 231 (6.0) | 1,419 (7.9) |
| Obesity | 28,374 (45.6) | 20,442 (46.3) | 7,705 (46.8) | 11,003 (46.2) | 1,734 (44.8) | 7,932 (43.9) |
| Anxiety | 9,936 (16.0) | 7,290 (16.5) | 2,658 (16.2) | 4,083 (17.1) | 549 (14.2) | 2,646 (14.6) |
| Depression | 10,909 (17.5) | 8,018 (18.2) | 2,940 (17.9) | 4,481 (18.8) | 597 (15.4) | 2,891 (16.0) |
| Asthma | 5,758 (9.3) | 4,238 (9.6) | 1,543 (9.4) | 2,357 (9.9) | 338 (8.7) | 1,520 (8.4) |
| Musculoskeletal pain | 27,579 (44.3) | 19,865 (45.0) | 7,342 (44.6) | 10,881 (45.6) | 1,642 (42.5) | 7,714 (42.7) |
| Osteoarthritis | 16,709 (26.9) | 11,903 (27.0) | 4,443 (27.0) | 6,560 (27.5) | 900 (23.3) | 4,806 (26.6) |
| **CCI adjusted without diabetes**, mean (SD) | 1.18 (1.56) | 1.14 (1.52) | 1.18 (1.55) | 1.13 (1.52) | 1.00 (1.44) | 1.27 (1.64) |
| **CCI components**, n (%) |  |  |  |  |  |  |
| Myocardial infarction | 4,340 (7.0) | 3,000 (6.8) | 1,176 (7.1) | 1,558 (6.5) | 266 (6.9) | 1,340 (7.4) |
| Congestive heart failure | 8,285 (13.3) | 5,521 (12.5) | 2,215 (13.5) | 2,822 (11.8) | 484 (12.5) | 2,764 (15.3) |
| COPD | 11,575 (18.6) | 8,277 (18.7) | 3,070 (18.7) | 4,574 (19.2) | 633 (16.4) | 3,298 (18.3) |
| Peripheral vascular disease | 9,216 (14.8) | 6,233 (14.1) | 2,483 (15.1) | 3,275 (13.7) | 475 (12.3) | 2,983 (16.5) |
| Cerebrovascular disease | 5,461 (8.8) | 3,721 (8.4) | 1,490 (9.1) | 1,922 (8.1) | 309 (8.0) | 1,740 (9.6) |
| Dementia | 1,110 (1.8) | 735 (1.7) | 279 (1.7) | 413 (1.7) | 43 (1.1) | 375 (2.1) |
| Rheumatic disease | 1,651 (2.7) | 1,170 (2.6) | 423 (2.6) | 672 (2.8) | 75 (1.9) | 481 (2.7) |
| Peptic ulcer disease | 618 (1.0) | 476 (1.1) | 172 (1.0) | 272 (1.1) | 32 (0.8) | 142 (0.8) |
| Mild liver disease | 6,052 (9.7) | 4,347 (9.8) | 1,659 (10.1) | 2,411 (10.1) | 277 (7.2) | 1,705 (9.4) |
| Hemiplegia or paraplegia | 515 (0.8) | 336 (0.8) | 147 (0.9) | 164 (0.7) | 25 (0.6) | 179 (1.0) |
| Renal disease | 11,840 (19.0) | 7,981 (18.1) | 3,000 (18.2) | 4,330 (18.2) | 651 (16.8) | 3,859 (21.4) |
| Any malignancy, including lymphoma and  leukemia, except malignant neoplasm  of skin | 4,170 (6.7) | 2,827 (6.4) | 1,098 (6.7) | 1,524 (6.4) | 205 (5.3) | 1,343 (7.4) |
| Moderate or severe liver disease | 323 (0.5) | 218 (0.5) | 86 (0.5) | 121 (0.5) | 11 (0.3) | 105 (0.6) |
| Metastatic solid tumor | 413 (0.7) | 260 (0.6) | 103 (0.6) | 137 (0.6) | 20 (0.5) | 153 (0.8) |
| AIDS/HIV | 4,149 (6.7) | 3,057 (6.9) | 1,109 (6.7) | 1,686 (7.1) | 262 (6.8) | 1,092 (6.0) |
| **DCSI**, mean (SD) | 1.72 (1.87) | 1.67 (1.84) | 1.74 (1.88) | 1.62 (1.79) | 1.67 (1.88) | 1.85 (1.93) |
| **DCSI components**, n (%) |  |  |  |  |  |  |
| Retinopathy | 10,177 (16.4) | 6,963 (15.8) | 2,760 (16.8) | 3,505 (14.7) | 698 (18.0) | 3,214 (17.8) |
| Nephropathy | 15,585 (25.0) | 10,601 (24.0) | 3,995 (24.3) | 5,682 (23.8) | 924 (23.9) | 4,984 (27.6) |
| Neuropathy | 17,001 (27.3) | 12,110 (27.4) | 4,796 (29.2) | 6,241 (26.2) | 1,073 (27.7) | 4,891 (27.1) |
| Cerebrovascular | 4,866 (7.8) | 3,324 (7.5) | 1,327 (8.1) | 1,717 (7.2) | 280 (7.2) | 1,542 (8.5) |
| Cardiovascular | 22,012 (35.4) | 15,054 (34.1) | 5,761 (35.0) | 8,043 (33.7) | 1,250 (32.3) | 6,958 (38.5) |
| Peripheral vascular disease | 10,845 (17.4) | 7,551 (17.1) | 2,925 (17.8) | 3,977 (16.7) | 649 (16.8) | 3,294 (18.2) |
| Metabolic | 2,386 (3.8) | 1,664 (3.8) | 653 (4.0) | 843 (3.5) | 168 (4.3) | 722 (4.0) |
| Tobacco smoking status | 6,884 (11.1) | 5,015 (11.4) | 1,870 (11.4) | 2,719 (11.4) | 426 (11.0) | 1,869 (10.3) |
| **Clinical characteristics and laboratory tests** |  |  |  |  |  |  |
| Latest HbA1c (%) during the baseline period,  mean (SD) | 8.63 (1.64) | 8.74 (1.67) | 8.67 (1.66) | 8.71 (1.66) | 9.26 (1.68) | 8.37 (1.52) |
| HbA1c ≥7% during the baseline period, n (%) | 57,427 (92.3) | 41,087 (93.0) | 15,170 (92.2) | 22,178 (93.0) | 3,739 (96.7) | 16,340 (90.4) |
| HbA1c ≥8% during the baseline period, n (%) | 36,338 (58.4) | 27,061 (61.3) | 9,754 (59.3) | 14,235 (59.7) | 3,072 (79.4) | 9,277 (51.4) |
| BMI (kg/m^2^) during the baseline period |  |  |  |  |  |  |
| <25, n (%) | 3,682 (5.9) | 2,526 (5.7) | 919 (5.6) | 1,393 (5.8) | 214 (5.5) | 1,156 (6.4) |
| 25-<30, n (%) | 12,224 (19.6) | 8,289 (18.8) | 3,088 (18.8) | 4,430 (18.6) | 771 (19.9) | 3,935 (21.8) |
| 30-<35, n (%) | 15,313 (24.6) | 10,762 (24.4) | 4,005 (24.3) | 5,811 (24.4) | 946 (24.5) | 4,551 (25.2) |
| 35-<40, n (%) | 10,754 (17.3) | 7,787 (17.6) | 2,820 (17.1) | 4,267 (17.9) | 700 (18.1) | 2,967 (16.4) |
| ≥40, n (%) | 6,270 (10.1) | 4,626 (10.5) | 1,721 (10.5) | 2,467 (10.3) | 438 (11.3) | 1,644 (9.1) |
| Unknown, n (%) | 13,979 (22.5) | 10,166 (23.0) | 3,896 (23.7) | 5,471 (22.9) | 799 (20.7) | 3,813 (21.1) |
| Kidney function and CKD stage, n (%) |  |  |  |  |  |  |
| Normal or CKD Stage 1 or CKD Stage 2 | 26,943 (43.3) | 19,266 (43.6) | 7,140 (43.4) | 10,354 (43.4) | 1,772 (45.8) | 7,677 (42.5) |
| Stage 3 | 10,444 (16.8) | 7,084 (16.0) | 2,617 (15.9) | 3,871 (16.2) | 596 (15.4) | 3,360 (18.6) |
| Stage 4 | 1,180 (1.9) | 715 (1.6) | 270 (1.6) | 369 (1.5) | 76 (2.0) | 465 (2.6) |
| Unknown | 23,655 (38.0) | 17,091 (38.7) | 6,422 (39.0) | 9,245 (38.8) | 1,424 (36.8) | 6,564 (36.3) |
| Time from the first observed T2D diagnosis  to the index date (months), mean (SD) | 72.83 (42.65) | 70.51 (41.54) | 70.63 (41.78) | 70.07 (41.81) | 72.74 (38.65) | 78.52 (44.76) |
| Lipid panel |  |  |  |  |  |  |
| LDL (mg/dL), mean (SD) | 86.56 (35.97) | 88.02 (36.47) | 88.13 (36.29) | 88.13 (36.72) | 86.86 (35.74) | 83.02 (34.47) |
| HDL (mg/dL), mean (SD) | 43.47 (12.28) | 43.51 (12.38) | 43.49 (12.28) | 43.63 (12.40) | 42.78 (12.70) | 43.39 (12.03) |
| VLDL (mg/dL), mean (SD) | 32.21 (23.89) | 32.73 (25.87) | 31.69 (17.76) | 33.41 (30.57) | 33.07 (23.04) | 30.96 (18.34) |
| Total cholesterol (mg/dL), mean (SD) | 164.74 (45.40) | 166.98 (46.19) | 166.50 (46.60) | 167.60 (46.36) | 165.19 (43.28) | 159.25 (42.90) |
| Triglycerides (mg/dL), mean (SD) | 196.34 (203.46) | 201.19 (220.38) | 198.41 (226.93) | 202.95 (220.32) | 202.10 (190.97) | 184.44 153.55) |
| C-reactive protein (mg/L), mean (SD) | 17.81 (39.27) | 17.45 (38.63) | 18.49 (41.69) | 16.71 (36.66) | 18.29 (38.27) | 18.74 (40.93) |
| **Vital signs** |  |  |  |  |  |  |
| Systolic blood pressure (mm Hg), mean (SD) | 131.10 (16.08) | 131.11 (16.12) | 131.37 (16.18) | 130.99 (16.05) | 130.77 (16.24) | 131.07 (16.00) |
| Diastolic blood pressure (mm Hg), mean (SD) | 76.84 (9.93) | 77.07 (9.96) | 77.12 (9.95) | 77.07 (9.95) | 76.89 (10.04) | 76.29 (9.85) |
| **Use of individual GLT classes during the baseline period**, n (%) | |  |  |  |  |  |
| Insulin | 14,629 (23.5) | 9,926 (22.5) | 4,486 (27.3) | 3,920 (16.4) | 1,520 (39.3) | 4,703 (26.0) |
| Thiazolidinediones | 4,678 (7.5) | 3,371 (7.6) | 1,342 (8.2) | 1,683 (7.1) | 346 (8.9) | 1,307 (7.2) |
| Sulfonylureas | 23,522 (37.8) | 16,859 (38.2) | 6,403 (38.9) | 8,531 (35.8) | 1,925 (49.8) | 6,663 (36.9) |
| Glucagon-like peptide receptor agonist | 13,031 (20.9) | 7,992 (18.1) | 3,881 (23.6) | 2,901 (12.2) | 1,210 (31.3) | 5,039 (27.9) |
| Biguanides | 47,914 (77.0) | 33,697 (76.3) | 13,142 (79.9) | 17,447 (73.2) | 3,108 (80.4) | 14,217 (78.7) |
| Meglitinides | 667 (1.1) | 480 (1.1) | 159 (1.0) | 265 (1.1) | 56 (1.4) | 187 (1.0) |
| Alpha-glucosidase inhibitors | 244 (0.4) | 194 (0.4) | 77 (0.5) | 96 (0.4) | 21 (0.5) | 50 (0.3) |
| Dipeptidyl peptidase 4 inhibitors | 13,457 (21.6) | 9,774 (22.1) | 3,518 (21.4) | 5,248 (22.0) | 1,008 (26.1) | 3,683 (20.4) |
| Number of GLT classes used during the baseline period, mean (SD) | 1.90 (1.03) | 1.86 (1.03) | 2.01 (1.03) | 1.68 (0.99) | 2.38 (1.02) | 1.98 (1.03) |
| Number of individual GLT used during the baseline period (generic name level), mean (SD) | 2.01 (1.20) | 1.97 (1.19) | 2.14 (1.22) | 1.76 (1.11) | 2.58 (1.23) | 2.11 (1.21) |
| **Selected non-GLT treatment use during the baseline period**, n (%) |  |  |  |  |  |  |
| Antihypertensive agents | 52,128 (83.8) | 36,494 (82.6) | 13,835 (84.1) | 19,330 (81.1) | 3,329 (86.1) | 15,634 (86.5) |
| Antiplatelets | 5,927 (9.5) | 4,062 (9.2) | 1,649 (10.0) | 2,045 (8.6) | 368 (9.5) | 1,865 (10.3) |
| Antihyperlipidemic agents | 49,647 (79.8) | 34,460 (78.0) | 13,147 (79.9) | 18,175 (76.2) | 3,138 (81.1) | 15,187 (84.1) |
| Anticoagulants | 5,468 (8.8) | 3,545 (8.0) | 1,330 (8.1) | 1,913 (8.0) | 302 (7.8) | 1,923 (10.6) |
| Antidepressants | 17,459 (28.1) | 12,670 (28.7) | 4,665 (28.4) | 6,985 (29.3) | 1,020 (26.4) | 4,789 (26.5) |
| Other HF medications except SGLT2i | 4,048 (6.5) | 2,750 (6.2) | 1,077 (6.5) | 1,408 (5.9) | 265 (6.9) | 1,298 (7.2) |
| **HCRU during the baseline period, mean (SD)** |  |  |  |  |  |  |
| Number of all-cause hospitalizations | 0.19 (0.65) | 0.18 (0.62) | 0.19 (0.64) | 0.18 (0.61) | 0.17 (0.58) | 0.21 (0.73) |
| Number of all-cause ED visits | 0.57 (1.66) | 0.58 (1.74) | 0.61 (1.80) | 0.56 (1.74) | 0.52 (1.44) | 0.54 (1.45) |
| Number of T2D-related ED visits | 0.18 (0.61) | 0.17 (0.58) | 0.18 (0.60) | 0.16 (0.56) | 0.16 (0.55) | 0.19 (0.70) |
| Number of T2D-related hospitalizations | 0.09 (0.49) | 0.10 (0.52) | 0.11 (0.48) | 0.09 (0.54) | 0.10 (0.51) | 0.09 (0.41) |
| **Cost during the baseline period, mean (SD) USD** |  |  |  |  |  |  |
| Costs of all-cause hospitalizations | 7,512.32 (33,298.05) | 7,193.49 (32,424.39) | 7,586.55 (33,582.38) | 7,062.10 (32,267.62) | 6,331.70 (28,067.46) | 8,291.61 (35,331.45) |
| Costs of all-cause ED visits | 273.87 (1,072.48) | 278.88 (1,136.64) | 303.23 (1,234.60) | 263.29 (967.69) | 271.41 (1,578.65) | 261.61 (896.47) |
| Costs of T2D-related ED visits | 6,042.91 (27,457.69) | 5,795.63 (26,843.06) | 6,100.49 (27,996.78) | 5,668.57 (26,400.89) | 5,282.24 (24,416.79) | 6,647.32 (28,896.84) |
| Costs of T2D-related hospitalizations | 49.41  (548.25) | 52.07 (608.50) | 58.43  (638.86) | 44.94  (418.32) | 68.95 (1,188.43) | 42.90 (360.85) |
| Out-of-pocket costs for all prescription drugs | 547.12  (856.03) | 527.05 (825.86) | 545.90  (857.08) | 496.14  (780.46) | 637.30  (942.82) | 596.19 (923.85) |

^a^Analysis 1 examined predictors the year before SGLT2i treatment initiation.

^b^Sample sizes smaller than 5 were reported as an aggregate (ie, <5) to adhere to the data vendor’s minimal sample size reporting requirements to protect individual privacy.

Abbreviations: AIDS/HIV, acquired immunodeficiency syndrome/human immunodeficiency virus; ASCVD, atherosclerotic cardiovascular disease; BMI, body mass index; CCI, Charlson Comorbidity Index; CKD, chronic kidney disease; COPD, chronic obstructive pulmonary disease; DCSI, Diabetes Complication Severity Index; DVT, deep vein thrombosis; ED, emergency department; GLT, glucose-lowering therapy; HbA1c, glycated hemoglobin; HCRU, health care resource utilization; HDL, high-density lipoprotein cholesterol; HF, heart failure; ICD-10, International Classification of Diseases, Tenth Revision; LDL, low-density lipoprotein cholesterol; MASH/MAFLD, metabolic dysfunction–associated steatohepatitis/metabolic dysfunction–associated fatty liver disease; PE, pulmonary embolism; SD, standard deviation; SGLT2i, sodium-glucose cotransporter-2 inhibitors; T2D, type 2 diabetes; USD, United States dollar; VLDL, very low-density lipoprotein cholesterol.

**Appendix Table 6.** Analysis 2: Baseline characteristics, overall and by treatment failure subtype.^a,b^

|  |  |  | **SGLT2i failure subtype** | | |  |
| --- | --- | --- | --- | --- | --- | --- |
| **Characteristics of individuals with T2D** | **Overall**  **sample N =62,222** | **SGLT2i failure overall n =44,156** | **Discontinuation group n =16,449** | **Failure with action group n = 23,839** | **Inertial failure group n = 3,868** | **Nonfailure group n = 18,066** |
| **Demographics** |  |  |  |  |  |  |
| Age at index date (years), mean (SD) | 62.65 (11.96) | 62.03 (12.01) | 62.19 (12.19) | 62.07 (11.93) | 61.12 (11.65) | 64.17 (11.72) |
| Age at index date, n (%) |  |  |  |  |  |  |
| 18-34 | 740 (1.2) | 568 (1.3) | 231 (1.4) | 291 (1.2) | 46 (1.2) | 172 (1.0) |
| 35-44 | 3,787 (6.1) | 2,937 (6.7) | 1,146 (7.0) | 1,544 (6.5) | 247 (6.4) | 850 (4.7) |
| 45-54 | 11,068 (17.8) | 8,359 (18.9) | 2,997 (18.2) | 4,522 (19.0) | 840 (21.7) | 2,709 (15.0) |
| 55-64 | 18,355 (29.5) | 13,058 (29.6) | 4,690 (28.5) | 7,132 (29.9) | 1,236 (32.0) | 5,297 (29.3) |
| 65-74 | 17,583 (28.3) | 12,193 (27.6) | 4,643 (28.2) | 6,586 (27.6) | 964 (24.9) | 5,390 (29.8) |
| ≥75 | 10,689 (17.2) | 7,041 (15.9) | 2,742 (16.7) | 3,764 (15.8) | 535 (13.8) | 3,648 (20.2) |
| Sex, n (%) |  |  |  |  |  |  |
| Male | 35,487 (57.0) | 24,380 (55.2) | 9,228 (56.1) | 12,856 (53.9) | 2,296 (59.4) | 11,107 (61.5) |
| Race, n (%) |  |  |  |  |  |  |
| Black | 7,725 (12.4) | 5,671 (12.8) | 2,289 (13.9) | 2,837 (11.9) | 545 (14.1) | 2,054 (11.4) |
| Asian | 2,668 (4.3) | 1,798 (4.1) | 741 (4.5) | 886 (3.7) | 171 (4.4) | 870 (4.8) |
| White | 44,774 (72.0) | 31,602 (71.6) | 11,356 (69.0) | 17,466 (73.3) | 2,780 (71.9) | 13,172 (72.9) |
| Other/unknown | 7,055 (11.3) | 5,085 (11.5) | 2,063 (12.5) | 2,650 (11.1) | 372 (9.6) | 1,970 (10.9) |
| Ethnicity, n (%) |  |  |  |  |  |  |
| Hispanic | 7,139 (11.5) | 5,205 (11.8) | 2,086 (12.7) | 2,719 (11.4) | 400 (10.3) | 1,934 (10.7) |
| Not Hispanic | 49,914 (80.2) | 35,258 (79.8) | 12,876 (78.3) | 19,173 (80.4) | 3,209 (83.0) | 14,656 (81.1) |
| Unknown | 5,169 (8.3) | 3,693 (8.4) | 1,487 (9.0) | 1,947 (8.2) | 259 (6.7) | 1,476 (8.2) |
| Index year, n (%) |  |  |  |  |  |  |
| 2015/2016 | 6,765 (10.9) | 5,592 (12.7) | 2,004 (12.2) | 2,961 (12.4) | <5 (<1.0) | 1,173 (6.5) |
| 2017 | 7,026 (11.3) | 5,700 (12.9) | 2,198 (13.4) | 2,840 (11.9) | 627 (16.2) | 1,326 (7.3) |
| 2018 | 8,067 (13.0) | 6,347 (14.4) | 2,439 (14.8) | 3,207 (13.5) | 662 (17.1) | 1,720 (9.5) |
| 2019 | 8,652 (13.9) | 6,693 (15.2) | 2,468 (15.0) | 3,556 (14.9) | 701 (18.1) | 1,959 (10.8) |
| 2020 | 10,915 (17.5) | 7,968 (18.0) | 2,916 (17.7) | 4,376 (18.4) | 669 (17.3) | 2,947 (16.3) |
| 2021 | 11,224 (18.0) | 7,214 (16.3) | 2,731 (16.6) | 4,016 (16.8) | 676 (17.5) | 4,010 (22.2) |
| 2022 | 8,802 (14.1) | 4,402 (10.0) | 1,637 (10.0) | 2,699 (11.3) | 467 (12.1) | 4,400 (24.4) |
| 2023 | 771 (1.2) | 240 (0.5) | 56 (0.3) | 184 (0.8) | 66 (1.7) | 531 (2.9) |
| Geographic region, n (%) |  |  |  |  |  |  |
| Midwest | 23,140 (37.2) | 16,453 (37.3) | 5,929 (36.0) | 8,985 (37.7) | 1,539 (39.8) | 6,687 (37.0) |
| Northeast | 11,949 (19.2) | 8,228 (18.6) | 3,126 (19.0) | 4,327 (18.2) | 775 (20.0) | 3,721 (20.6) |
| South | 17,616 (28.3) | 12,717 (28.8) | 4,832 (29.4) | 738 (3.1) | 111 (2.9) | 492 (2.7) |
| West | 7,687 (12.4) | 5,420 (12.3) | 2,073 (12.6) | 6,896 (28.9) | 989 (25.6) | 4,899 (27.1) |
| Other/unknown | 1,830 (2.9) | 1,338 (3.0) | 489 (3.0) | 2,893 (12.1) | 454 (11.7) | 2,267 (12.5) |
| Payer, n (%) |  |  |  |  |  |  |
| Commercial | 27,378 (44.0) | 19,800 (44.8) | 7,017 (42.7) | 10,847 (45.5) | 1,936 (50.1) | 7,578 (41.9) |
| Medicaid | 4,127 (6.6) | 3,042 (6.9) | 1,253 (7.6) | 1,529 (6.4) | 260 (6.7) | 1,085 (6.0) |
| Medicare Advantage or Medicare | 29,882 (48.0) | 20,753 (47.0) | 7,954 (48.4) | 11,167 (46.8) | 1,632 (42.2) | 9,129 (50.5) |
| Other/unknown | 835 (1.3) | 561 (1.3) | 225 (1.4) | 296 (1.2) | 40 (1.0) | 274 (1.5) |
| **Comorbidities**, n (%) |  |  |  |  |  |  |
| Atrial fibrillation | 4,947 (8.0) | 3,354 (7.6) | 1,268 (7.7) | 1,757 (7.4) | 329 (8.5) | 1,593 (8.8) |
| Ischemic heart disease | 13,742 (22.1) | 9,624 (21.8) | 3,715 (22.6) | 4,904 (20.6) | 1,005 (26.0) | 4,118 (22.8) |
| DVT and PE | 953 (1.5) | 683 (1.5) | 249 (1.5) | 367 (1.5) | 67 (1.7) | 270 (1.5) |
| Hypertension | 42,868 (68.9) | 31,053 (70.3) | 11,770 (71.6) | 16,128 (67.7) | 3,155 (81.6) | 11,815 (65.4) |
| Hyperlipidemia/dyslipidemia | 41,824 (67.2) | 30,372 (68.8) | 11,333 (68.9) | 15,842 (66.5) | 3,197 (82.7) | 11,452 (63.4) |
| ASCVD components including |  |  |  |  |  |  |
| Ischemic stroke | 1,344 (2.2) | 947 (2.1) | 366 (2.2) | 467 (2.0) | 114 (2.9) | 397 (2.2) |
| Transient ischemic attack | 664 (1.1) | 486 (1.1) | 186 (1.1) | 248 (1.0) | 52 (1.3) | 178 (1.0) |
| Other atherosclerotic  cerebrovascular disease | 3,146 (5.1) | 2,220 (5.0) | 898 (5.5) | 1092 (4.6) | 230 (5.9) | 926 (5.1) |
| Myocardial infarction | 1,229 (2.0) | 863 (2.0) | 337 (2.0) | 435 (1.8) | 91 (2.4) | 366 (2.0) |
| Other coronary heart disease | 12,411 (19.9) | 8,694 (19.7) | 3,345 (20.3) | 4,424 (18.6) | 925 (23.9) | 3,717 (20.6) |
| Peripheral artery disease | 5,433 (8.7) | 3,921 (8.9) | 1,643 (10.0) | 1,880 (7.9) | 398 (10.3) | 1,512 (8.4) |
| MASH/MAFLD | 3,400 (5.5) | 2,548 (5.8) | 936 (5.7) | 1,389 (5.8) | 223 (5.8) | 852 (4.7) |
| Obesity | 21,224 (34.1) | 15,808 (35.8) | 5,816 (35.4) | 8,327 (34.9) | 1,665 (43.0) | 5,416 (30.0) |
| Anxiety | 7,258 (11.7) | 5,478 (12.4) | 2,037 (12.4) | 2,913 (12.2) | 528 (13.7) | 1,780 (9.9) |
| Depression | 8,171 (13.1) | 6,202 (14.0) | 2,319 (14.1) | 3,263 (13.7) | 620 (16.0) | 1,969 (10.9) |
| Asthma | 3,961 (6.4) | 3,002 (6.8) | 1,136 (6.9) | 1,566 (6.6) | 300 (7.8) | 959 (5.3) |
| Musculoskeletal pain | 20,143 (32.4) | 14,915 (33.8) | 5,723 (34.8) | 7,614 (31.9) | 1,578 (40.8) | 5,228 (28.9) |
| Osteoarthritis | 12,189 (19.6) | 8,906 (20.2) | 3,442 (20.9) | 4,586 (19.2) | 878 (22.7) | 3,283 (18.2) |
| **CCI adjusted without diabetes**, mean (SD) | 0.96 (1.46) | 0.96 (1.45) | 1.01 (1.49) | 0.91 (1.41) | 1.07 (1.46) | 0.95 (1.49) |
| **CCI components**, n (%) |  |  |  |  |  |  |
| Myocardial infarction | 2,980 (4.8) | 2,136 (4.8) | 831 (5.1) | 1,073 (4.5) | 232 (6.0) | 844 (4.7) |
| Congestive heart failure | 7,034 (11.3) | 4,841 (11.0) | 1,910 (11.6) | 2,421 (10.2) | 510 (13.2) | 2,193 (12.1) |
| COPD | 8,465 (13.6) | 6,231 (14.1) | 2,359 (14.3) | 3,240 (13.6) | 632 (16.3) | 2,234 (12.4) |
| Peripheral vascular disease | 7,335 (11.8) | 5,222 (11.8) | 2,175 (13.2) | 2,537 (10.6) | 510 (13.2) | 2,113 (11.7) |
| Cerebrovascular disease | 4,132 (6.6) | 2,947 (6.7) | 1,154 (7.0) | 1,488 (6.2) | 305 (7.9) | 1,185 (6.6) |
| Dementia | 1,040 (1.7) | 751 (1.7) | 296 (1.8) | 397 (1.7) | 58 (1.5) | 289 (1.6) |
| Rheumatic disease | 1,280 (2.1) | 947 (2.1) | 366 (2.2) | 500 (2.1) | 81 (2.1) | 333 (1.8) |
| Peptic ulcer disease | 426 (0.7) | 311 (0.7) | 121 (0.7) | 164 (0.7) | 26 (0.7) | 115 (0.6) |
| Mild liver disease | 4,186 (6.7) | 3,104 (7.0) | 1,162 (7.1) | 1,673 (7.0) | 269 (7.0) | 1,082 (6.0) |
| Hemiplegia or paraplegia | 404 (0.6) | 295 (0.7) | 121 (0.7) | 145 (0.6) | 29 (0.7) | 109 (0.6) |
| Renal disease | 11,029 (17.7) | 7,791 (17.6) | 2,931 (17.8) | 4,087 (17.1) | 773 (20.0) | 3,238 (17.9) |
| Any malignancy, including lymphoma and  leukemia, except malignant neoplasm of  skin | 3,561 (5.7) | 2,510 (5.7) | 1,006 (6.1) | 1,287 (5.4) | 217 (5.6) | 1,051 (5.8) |
| Moderate or severe liver disease | 300 (0.5) | 198 (0.4) | 82 (0.5) | 96 (0.4) | 20 (0.5) | 102 (0.6) |
| Metastatic solid tumor | 442 (0.7) | 300 (0.7) | 126 (0.8) | 152 (0.6) | 22 (0.6) | 142 (0.8) |
| AIDS/HIV | 4,028 (6.5) | 3,211 (7.3) | 1,189 (7.2) | 1,734 (7.3) | 288 (7.4) | 817 (4.5) |
| **DCSI,** mean (SD) | 1.45 (1.76) | 1.46 (1.75) | 1.53 (1.79) | 1.36 (1.69) | 1.78 (1.84) | 1.43 (1.78) |
| **DCSI components**, n (%) |  |  |  |  |  |  |
| Retinopathy | 8,171 (13.1) | 5,868 (13.3) | 2,339 (14.2) | 2,752 (11.5) | 777 (20.1) | 2,303 (12.7) |
| Nephropathy | 14,566 (23.4) | 10,338 (23.4) | 3,903 (23.7) | 5,385 (22.6) | 1,050 (27.1) | 4,228 (23.4) |
| Neuropathy | 14,266 (22.9) | 10,494 (23.8) | 4,151 (25.2) | 5,189 (21.8) | 1,154 (29.8) | 3,772 (20.9) |
| Cerebrovascular | 3,620 (5.8) | 2,581 (5.8) | 1,000 (6.1) | 1,315 (5.5) | 266 (6.9) | 1,039 (5.8) |
| Cardiovascular | 18,746 (30.1) | 13,222 (29.9) | 5,095 (31.0) | 6,782 (28.4) | 1,345 (34.8) | 5,524 (30.6) |
| Peripheral vascular disease | 9,294 (14.9) | 6,706 (15.2) | 2,679 (16.3) | 3,353 (14.1) | 674 (17.4) | 2,588 (14.3) |
| Metabolic | 1,805 (2.9) | 1,338 (3.0) | 505 (3.1) | 662 (2.8) | 171 (4.4) | 467 (2.6) |
| Tobacco smoking status | 4,707 (7.6) | 3,507 (7.9) | 1,298 (7.9) | 1,843 (7.7) | 366 (9.5) | 1,200 (6.6) |
| **Clinical characteristics and laboratory tests** |  |  |  |  |  |  |
| Latest HbA1c closest to failure date | 7.95 (1.50) | 8.16 (1.54) | 7.62 (1.42) | 8.39 (1.55) | 8.86 (1.37) | 7.39 (1.18) |
| HbA1c ≥7% closest to failure date, n (%) | 33,297 (53.5) | 25,987 (58.9) | 7,821 (47.5) | 14,395 (60.4) | 3771 (97.5) | 7,310 (40.5) |
| HbA1c ≥8% closest to failure date, n (%) | 18,315 (29.4) | 15,680 (35.5) | 3,465 (21.1) | 8,938 (37.5) | 3277 (84.7) | 2,635 (14.6) |
| BMI (kg/m^2^) 1 year before failure date |  |  |  |  |  |  |
| <25, n (%) | 4,287 (6.9) | 2,965 (6.7) | 1,162 (7.1) | 1,487 (6.2) | 316 (8.2) | 1,322 (7.3) |
| 25-<30, n (%) | 11,770 (18.9) | 8,257 (18.7) | 3,234 (19.7) | 4,171 (17.5) | 852 (22.0) | 3,513 (19.4) |
| 30-<35, n (%) | 13,088 (21.0) | 9,392 (21.3) | 3,478 (21.1) | 4,922 (20.6) | 992 (25.6) | 3,696 (20.5) |
| 35-<40, n (%) | 8,610 (13.8) | 6,404 (14.5) | 2,343 (14.2) | 3,371 (14.1) | 690 (17.8) | 2,206 (12.2) |
| ≥40, n (%) | 4,660 (7.5) | 3,502 (7.9) | 1,346 (8.2) | 1,796 (7.5) | 360 (9.3) | 1,158 (6.4) |
| Unknown, n (%) | 19,807 (31.8) | 13,636 (30.9) | 4,886 (29.7) | 8,092 (33.9) | 658 (17.0) | 6,171 (34.2) |
| Kidney function and CKD stage, n (%) |  |  |  |  |  |  |
| Normal or CKD Stage1 or CKD Stage 2 | 12,885 (20.7) | 9,735 (22.0) | 3,270 (19.9) | 5,017 (21.0) | 1448 (37.4) | 3,150 (17.4) |
| Stage 3 | 8,574 (13.8) | 6,127 (13.9) | 2,206 (13.4) | 3,235 (13.6) | 686 (17.7) | 2,447 (13.5) |
| Stage 4 | 1,201 (1.9) | 772 (1.7) | 299 (1.8) | 393 (1.6) | 80 (2.1) | 429 (2.4) |
| Stage 5 | 156 (0.3) | 114 (0.3) | 50 (0.3) | 47 (0.2) | 17 (0.4) | 42 (0.2) |
| Unknown | 39,406 (63.3) | 27,408 (62.1) | 10,624 (64.6) | 15,147 (63.5) | 1637 (42.3) | 11,998 (66.4) |
| Time from the first observed T2D diagnosis to the index date (months), mean (SD) | 72.83 (42.65) | 70.51 (41.54) | 70.63 (41.78) | 70.07 (41.81) | 72.74 (38.65) | 78.52 (44.76) |
| Lipid panel |  |  |  |  |  |  |
| LDL (mg/dL), mean (SD) | 84.09 (35.12) | 85.54 (35.80) | 84.44 (35.42) | 86.20 (35.91) | 85.97 (36.36) | 80.12 (32.84) |
| HDL (mg/dL), mean (SD) | 44.58 (12.52) | 44.44 (12.43) | 44.67 (12.33) | 44.57 (12.67) | 43.27 (11.71) | 44.97 (12.74) |
| VLDL (mg/dL), mean (SD) | 31.00 (19.13) | 31.71 (19.99) | 30.13 (17.93) | 32.38 (20.60) | 33.58 (22.74) | 29.08 (16.42) |
| Total cholesterol (mg/dL), mean (SD) | 161.49 (43.60) | 163.87 (44.67) | 160.86 (43.64) | 165.68 (45.61) | 164.95 (43.22) | 154.91 (39.77) |
| Triglycerides (mg/dL), mean (SD) | 187.49 (159.54) | 194.26 (171.17) | 178.29 (147.53) | 202.54 (183.47) | 205.49 (177.93) | 168.65 (119.50) |
| C-reactive protein (mg/L), mean (SD) | 18.83 (42.07) | 19.06 (40.80) | 17.72 (40.22) | 19.25 (39.58) | 22.40 (47.26) | 18.13 (45.63) |
| **Vital signs** |  |  |  |  |  |  |
| Systolic blood pressure (mm Hg), mean (SD) | 128.25 (15.54) | 128.46 (15.58) | 128.40 (15.88) | 128.42 (15.34) | 128.90 (15.65) | 127.70 (15.41) |
| Diastolic blood pressure (mm Hg), mean (SD) | 75.24 (9.80) | 75.50 (9.84) | 75.16 (9.86) | 75.74 (9.77) | 75.57 (10.04) | 74.59 (9.68) |
| **Use of individual GLT classes 1 year before outcome date**, n (%) |  |  |  |  |  |  |
| Insulin | 11,853 (19.0) | 8,191 (18.6) | 3,797 (23.1) | 2,981 (12.5) | 1,413 (36.5) | 3,662 (20.3) |
| Thiazolidinediones | 2,673 (4.3) | 1,925 (4.4) | 829 (5.0) | 895 (3.8) | 201 (5.2) | 748 (4.1) |
| Sulfonylureas | 16,423 (26.4) | 12,016 (27.2) | 4,729 (28.7) | 5,784 (24.3) | 1,503 (38.9) | 4,407 (24.4) |
| Glucagon-like peptide receptor agonist | 9,290 (14.9) | 5,674 (12.8) | 2,929 (17.8) | 1,794 (7.5) | 951 (24.6) | 3,616 (20.0) |
| Biguanides | 38,494 (61.9) | 27,554 (62.4) | 11,238 (68.3) | 13,506 (56.7) | 2,810 (72.6) | 10,940 (60.6) |
| Meglitinides | 367 (0.6) | 261 (0.6) | 94 (0.6) | 134 (0.6) | 33 (0.9) | 106 (0.6) |
| Alpha-glucosidase inhibitors | 119 (0.2) | 90 (0.2) | 35 (0.2) | 41 (0.2) | 14 (0.4) | 29 (0.2) |
| Dipeptidyl peptidase 4 inhibitors | 8,157 (13.1) | 5,992 (13.6) | 2,188 (13.3) | 3,183 (13.4) | 621 (16.1) | 2,165 (12.0) |
| Number of GLT classes used, mean (SD) | 2.35 (1.01) | 2.36 (0.97) | 2.57 (0.92) | 2.14 (0.93) | 2.83 (1.00) | 2.32 (1.10) |
| Number of individual GLT used  (generic name level), Mean (SD) | 2.44 (1.14) | 2.46 (1.10) | 2.69 (1.08) | 2.20 (1.03) | 3.05 (1.20) | 2.41 (1.23) |
| **Selected non-GLT treatment use 1 year before outcome date**, n (%) |  |  |  |  |  |  |
| Antihypertensive agents | 48,507 (78.0) | 34,705 (78.6) | 13,500 (82.1) | 17,866 (74.9) | 3,339 (86.3) | 13,802 (76.4) |
| Antiplatelets | 5,350 (8.6) | 3,759 (8.5) | 1,568 (9.5) | 1,787 (7.5) | 404 (10.4) | 1,591 (8.8) |
| Antihyperlipidemic agents | 46,155 (74.2) | 33,076 (74.9) | 12,850 (78.1) | 16,944 (71.1) | 3,282 (84.9) | 13,079 (72.4) |
| Anticoagulants | 5,358 (8.6) | 3,607 (8.2) | 1,383 (8.4) | 1,888 (7.9) | 336 (8.7) | 1,751 (9.7) |
| Antidepressants | 15,630 (25.1) | 11,615 (26.3) | 4,404 (26.8) | 6,144 (25.8) | 1,067 (27.6) | 4,015 (22.2) |
| Other HF medications except SGLT2i | 3,759 (6.0) | 2,558 (5.8) | 1,040 (6.3) | 1,270 (5.3) | 248 (6.4) | 1,201 (6.6) |
| **HCRU 1 year before outcome date**, mean (SD) |  |  |  |  |  |  |
| Number of all-cause hospitalizations | 0.11 (0.50) | 0.12 (0.50) | 0.13 (0.54) | 0.11 (0.46) | 0.14 (0.55) | 0.10 (0.52) |
| Number of all-cause ED visits | 0.33 (1.19) | 0.35 (1.24) | 0.36 (1.33) | 0.32 (1.16) | 0.45 (1.35) | 0.28 (1.06) |
| Number of all ED visits | 0.11 (0.48) | 0.11 (0.48) | 0.12 (0.51) | 0.10 (0.45) | 0.13 (0.53) | 0.10 (0.50) |
| Number of T2D-related ED visits | 0.05 (0.36) | 0.06 (0.39) | 0.06 (0.35) | 0.06 (0.42) | 0.07 (0.36) | 0.04 (0.29) |
| **Cost 1 year before outcome date**, mean (SD) USD |  |  |  |  |  |  |
| Costs of all-cause hospitalizations | 4,700.42 (28,557.64) | 4,887.47 (27,985.36) | 5,394.54 (31,037.34) | 4,539.62 (26,297.33) | 4,874.99 (24,091.52) | 4,243.24 (29,906.21) |
| Costs of all-cause ED visits | 162.02  (789.35) | 173.09 (845.20) | 188.86 (1,015.78) | 154.84  (655.31) | 218.46 (1,056.32) | 134.96 (631.61) |
| Costs of T2D-related ED visits | 3,826.20 (23,853.33) | 4,015.15 (23,695.04) | 4,351.69 (25,630.94) | 3,767.17 (22,649.04) | 4,112.30 (21,296.53) | 3,364.38 (24,230.31) |
| Costs of T2D-related hospitalizations | 27.46  (364.30) | 31.45 (400.76) | 31.00  (386.13) | 30.55  (379.17) | 38.99  (559.83) | 17.70 (253.78) |
| Out-of-pocket costs for all prescription drugs | 556.51 (866.97) | 570.47 (879.68) | 609.45  (944.17) | 506.71  (796.22) | 797.71 (1,026.18) | 522.37 (834.12) |
| **Potential clinical events relevant to SGLT2i failure 1 year before the outcome date** **(only in Analysis 2),** n (%) |  |  |  |  |  |  |
| Experienced a urinary tract infection | 5,911 (9.5) | 4,507 (10.2) | 1,688 (10.3) | 2,517 (10.6) | 302 (7.8) | 1,404 (7.8) |
| Experienced a urinary tract infection leading  to ED or hospitalization | 1,109 (1.8) | 840 (1.9) | 345 (2.1) | 433 (1.8) | 62 (1.6) | 269 (1.5) |
| Experienced a genital mycotic infection | 1,636 (2.6) | 1,394 (3.2) | 496 (3.0) | 800 (3.4) | 98 (2.5) | 242 (1.3) |
| Experienced hypoglycemia | 1,685 (2.7) | 1,191 (2.7) | 487 (3.0) | 572 (2.4) | 132 (3.4) | 494 (2.7) |
| Experienced hypoglycemia leading to ED or  hospitalization | 317 (0.5) | 231 (0.5) | 93 (0.6) | 98 (0.4) | 40 (1.0) | 86 (0.5) |
| Experienced a lower limb amputation | 203 (0.3) | 148 (0.3) | 56 (0.3) | 88 (0.4) | <5 (<1.0) | 55 (0.3) |
| Experienced a Fournier's gangrene | 46 (0.1) | 40 (0.1) | 16 (0.1) | 23 (0.1) | <5 (<1.0) | 6 (<0.1) |
| Pre-post changes in HbA1c (%):  (Post-index HbA1c) - (Pre-index HbA1c), mean (SD) | -0.67 (1.71) | -0.56 (1.76) | -1.01 (1.71) | -0.27 (1.72) | -0.40 (1.75) | -0.96 (1.55) |
| Pre-post changes in eGFR (mL/min/1.73 m²), n (%) |  |  |  |  |  |  |
| Decline ≥40% | 522 (0.8) | 393 (0.9) | 121 (0.7) | 232 (1.0) | 40 (1.0) | 129 (0.7) |
| Decline 0-<40% | 22,858 (36.7) | 16,747 (37.9) | 6,053 (36.8) | 8,674 (36.4) | 2,020 (52.2) | 6,111 (33.8) |
| Increase | 13,704 (22.0) | 9,987 (22.6) | 3,738 (22.7) | 5,137 (21.5) | 1,112 (28.7) | 3,717 (20.6) |
| Unknown | 25,138 (40.4) | 17,029 (38.6) | 6,537 (39.7) | 9,796 (41.1) | 696 (18.0) | 8,109 (44.9) |

^a^Analysis 2 examined predictors the year before treatment failure with SGLT2i.

^b^Sample sizes smaller than 5 were reported as an aggregate (ie, <5) to adhere to the data vendor’s minimal sample size reporting requirements to protect individual privacy.

Abbreviations: AIDS/HIV, acquired immunodeficiency syndrome/human immunodeficiency virus; ASCVD, atherosclerotic cardiovascular disease; BMI, body mass index; CCI, Charlson Comorbidity Index; CKD, chronic kidney disease; COPD, chronic obstructive pulmonary disease; DCSI, Diabetes Complication Severity Index; DVT, deep vein thrombosis; ED, emergency department; eGFR, estimated glomerular filtration rate; GLT, glucose-lowering therapy; HbA1c, glycated hemoglobin; HCRU, health care resource utilization; HDL, high-density lipoprotein cholesterol; HF, heart failure; ICD-10, International Classification of Diseases, Tenth Revision; LDL, low-density lipoprotein cholesterol; MASH/MAFLD, metabolic dysfunction–associated steatohepatitis/metabolic dysfunction–associated fatty liver disease; PE, pulmonary embolism; SD, standard deviation; SGLT2i, sodium-glucose cotransporter-2 inhibitors; T2D, type 2 diabetes; USD, United States dollar; VLDL, very low-density lipoprotein cholesterol.

**Appendix Figure 1.** Study sample selection flow diagram.

| Newly initiated any approved SGLT2i treatments (ie, bexagliflozin, canagliflozin, dapagliflozin, empagliflozin, ertugliflozin) from December 31, 2016, through April 3, 2024. The date of the first eligible SGLT2i pharmacy claim was defined as the index date. Individuals must have had no SGLT2i use prior to the index date. |
| --- |
| **N = 1,895,040** |
|  |
| Individuals who had ≥2 diagnoses of T2D on ≥2 distinct days  in the study period (January 1, 2016—September 30, 2024). |
| **N = 1,658,627** |
|  |
| Individuals who had the first T2D diagnosis on or before the index date. |
| **N = 1,577,987** |
|  |
| Individuals with continuous claims enrollment in the predictor lookup period and outcome assessment period. The intervals prior to the index date (in Analysis 1) or outcome date (in Analysis 2) are defined as the predictor lookup period, which is required to be at least one year. The interval from the index date to the end of follow-up is referred to as the outcome assessment period, which needs to be at least 180 days. |
| **N = 526,013** |
|  |
| Individuals who were aged ≥18 at the index date. |
| **N = 525,896** |
|  |
| Individuals who had ≥1 HbA1c value ≥7% during the baseline period or on the index date. |
| **N = 117,050** |
|  |
| Individuals who had ≥1 HbA1c value from 180 days after index until the end of follow-up. |
| **N = 80,036** |
|  |
| Individuals who had no diagnosis of T1D during the study period. |
| **N = 66,040** |
|  |
| Individuals who had nonmissing sex |
| **N = 64,929** |
|  |
| Individuals who did not initiate another GLT drug class on the index date or used any GLP-1 RA obesity products (Wegovy [semaglutide], Saxenda [liraglutide], Zepbound [tirzepatide]) during the study period. |
| **N = 62,880** |
|  |
| Individuals who did not have evidence of pregnancy during the baseline or follow-up period. |
| **N = 62,529** |
|  |
| Individuals who did not have evidence of CKD stage 5 or ESKD during baseline or on the index date, identified by ICD codes (ICD-10: N18.5, N18.6), procedure codes for dialysis and kidney transplant, as well as eGFR values (required to have two eGFR values <15 which are 90-365 days apart). |
| **N = 62,222**  **(Final Study Sample)** |
| Abbreviations: CKD, chronic kidney disease; eGFR, estimated glomerular filtration rate; ESKD, end-stage kidney disease; GLP-1 RA, glucagon-like peptide-1 receptor agonist; GLT, glucose-lowering therapy; HbA1c, glycated hemoglobin; ICD-10, International Classification of Diseases, Tenth Revision; SGLT2i, sodium-glucose cotransporter-2 inhibitors; T1D, type 1 diabetes; T2D, type 2 diabetes. |

**Appendix Figure 2.** Data preparation and processing diagram.

**
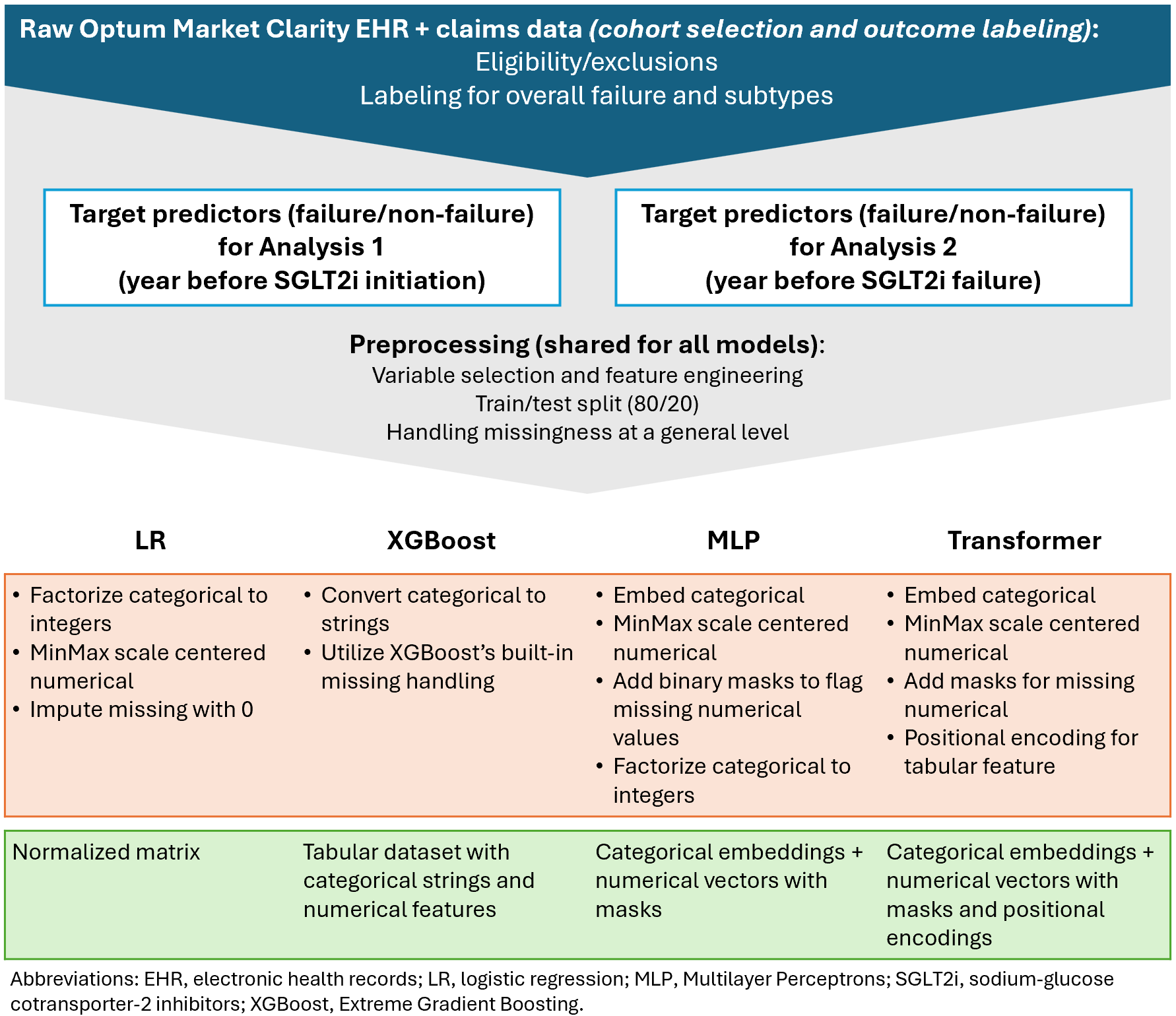
**
